# Supplementary material for: Efficient, narrow-band, and stable electroluminescence from organoboron-nitrogen-carbonyl emitter
Source: Nat Commun. 2024 Jan 25;15:731. doi: 10.1038/s41467-024-44981-1 (PMC10810797; doi:10.1038/s41467-024-44981-1)
Supplement: Supplementary file 1 — Supplementary Information [file 41467_2024_44981_MOESM1_ESM.pdf]

Efficient, narrow-band, and stable electroluminescence from organoboron-nitrogen-carbonyl emitter

Ying-Chun Cheng[1,6], Xun Tang[2,6], Kai Wang[1,3]*, Xin Xiong[1], Xiao-Chun Fan[1], Shulin Luo[1], Rajat Walia[1], Yue Xie[1], Tao Zhang[1], Dandan Zhang[1], Jia Yu[1,4], Xian-Kai Chen[1,*], Chihaya Adachi[2,5,*], and Xiao-Hong Zhang[1,4,*]

[1]Institute of Functional Nano & Soft Materials (FUNSOM), Joint International Research Laboratory of Carbon-Based Functional Materials and Devices, Soochow University, Suzhou, Jiangsu 215123, PR China.
[2]Center for Organic Photonics and Electronics Research (OPERA), Kyushu University, 744 Motooka, Nishi-ku, Fukuoka, 819-0395, Japan.
[3]Jiangsu Key Laboratory for Carbon-Based Functional Materials & Devices, Soochow University, Suzhou, 215123, Jiangsu, PR China
[4]Jiangsu Key Laboratory of Advanced Negative Carbon Technologies, Soochow University, Suzhou, 215123, Jiangsu, PR China
[5]International Institute for Carbon-Neutral Energy Research (I2CNER), Kyushu University, 744 Motooka, Nishi, Fukuoka 819-0395, Japan.
[6]These authors contributed equally: Y.-C. C., X. T.

*e-mail: wkai@suda.edu.cn (K.W.), xkchen@suda.edu.cn (X.-K.C.), adachi@cstf.kyushu-u.ac.jp (C.A.), xiaohong_zhang@suda.edu.cn (X.-H.Z.)

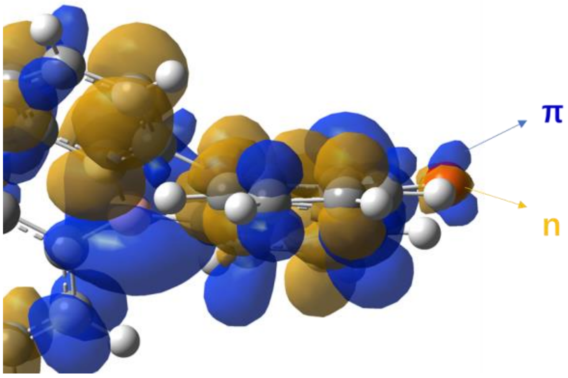

Supplementary Fig. 1. Calculated difference-density plot (side view) of the T_1 excited state for ***h*-BNCO-1** via the high-level STEOM-DLPNO-CCSD method.

Supplementary Table 1. Calculated electronic-structure parameters of ***h*-BNCO-1**, BNCZ, and BNBNB. E_{S1} / E_{T1} / E_{T2} denotes the excitation energy of the S_1 / T_1 / T_2 state, as calculated in the STEOM-DLPNO-CCSD/def2-TZVP theory level; *SOC*(T_1-S_1) / *SOC*(T_2-S_1) denotes the spin-orbit coupling between the S_1 and T_1 / T_2 states, as calculated in the STEOM-DLPNO-CCSD/def2-TZVP theory level; $\lambda_{T1\rightarrow S1}$ / $\lambda_{T2\rightarrow S1}$ denotes the reorganization energy related to the transition from T_1 / T_2 to S_1, as calculated in the ωB97XD/6-31 G(d,p) theory level.

	E_{S1} / eV	E_{T1} / eV	E_{T2} / eV	*SOC*(T_1-S_1) / cm^{-1}	*SOC*(T_2-S_1) / cm^{-1}	$\lambda_{T1\rightarrow S1}$ / eV	$\lambda_{T2\rightarrow S1}$ / eV
h-BNCO-1	2.65	2.53	2.71	0.04	1.46	0.0078	0.08
BNCZ	2.81	2.61	2.87	0.07	0.29	0.042	0.346
BNBNB	2.56	2.48	2.66	0.01	0.69	0.0057	0.029

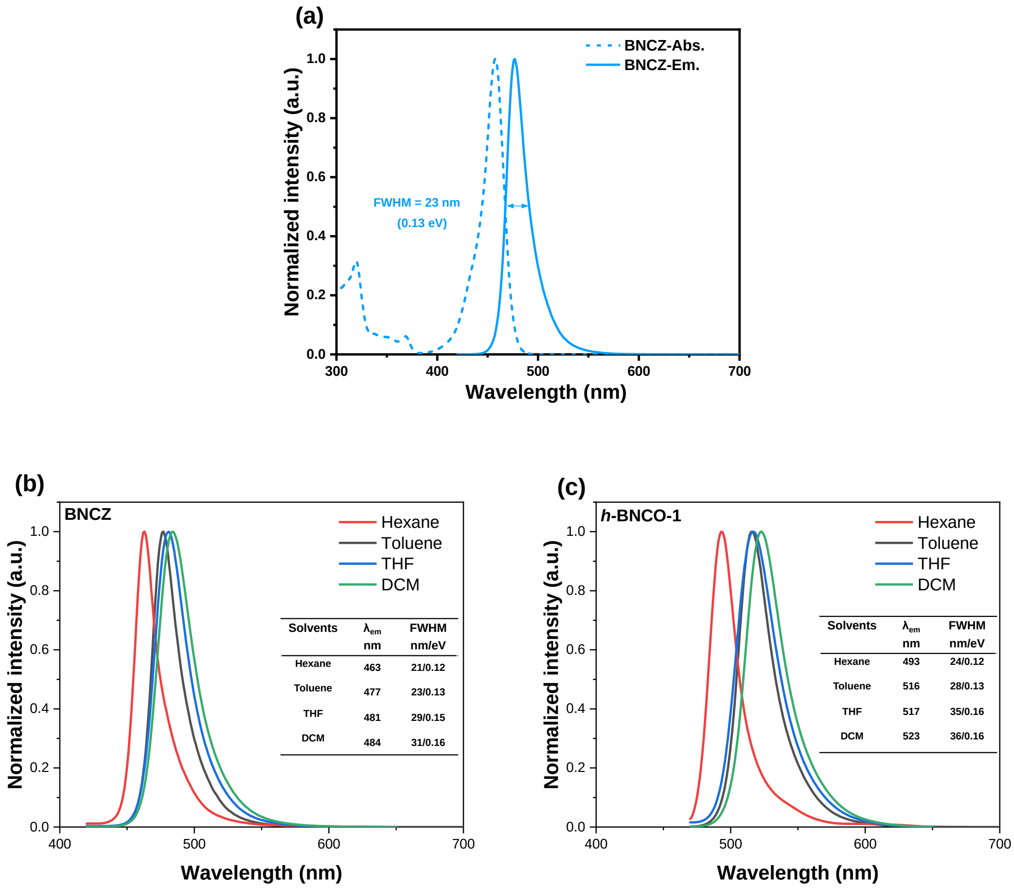

Supplementary Fig. 2. (a) Normalized absorption and emission spectra of BNCZ in toluene solution (1×10^{-5} M) at room temperature; normalized emission spectra of (b) BNCZ and (c) *h*-BNCO-1 in various solvents (1×10^{-5} M) at room temperature.

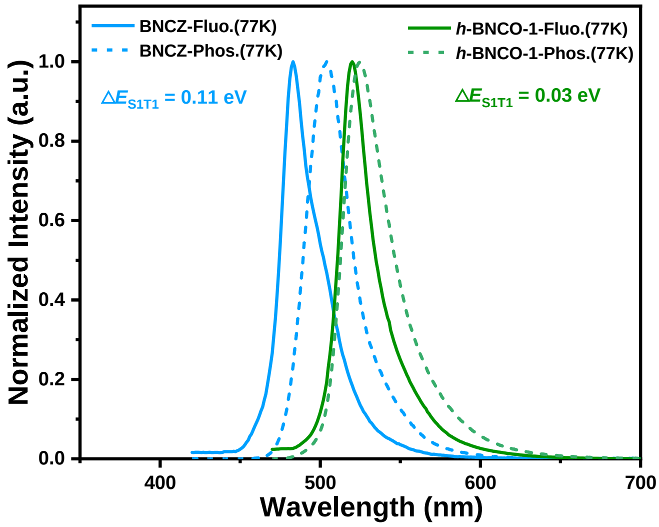

Supplementary Fig. 3. Fluorescence and phosphorescence spectra of BNCZ and *h*-BNCO-1 in toluene solution (1×10^{-5} M) at 77 K.

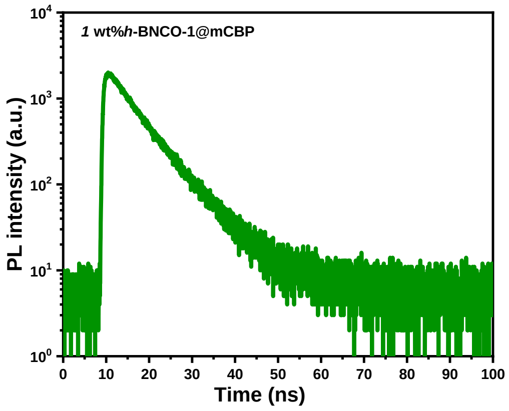

Supplementary Fig. 4. Transient PL decays of ***h*-BNCO-1** 1 wt% doped in mCBP film measured in a nanosecond scale time range.

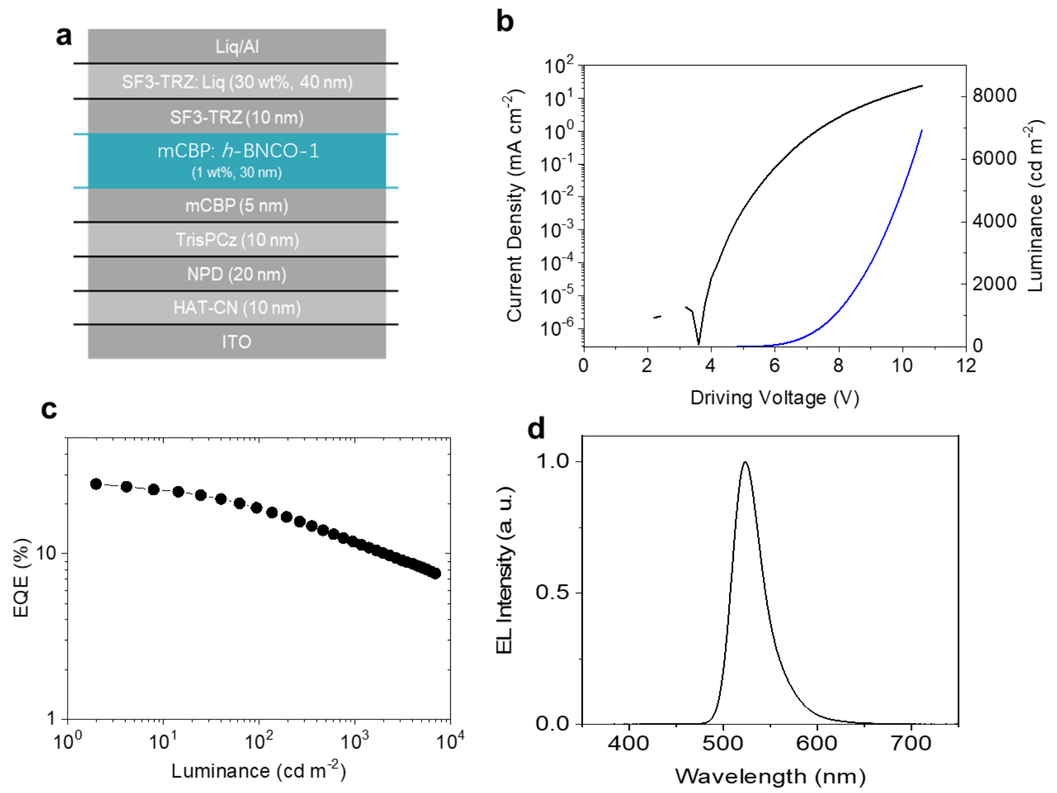

Supplementary Fig. 5. OLED performance based on the EML of 1 wt% *h*-BNCO-1: mCBP. **a**, Device structure. **b**, Current density−driving voltage−luminance (J−V−L) characteristics. **c**, EQE-to-luminance curve. **d**, EL spectrum at 1000 cd m^{-2}. The maximum EQE was around 26.3%, and EQE at 1000 cd m^{-2} was about 11.5%. The relatively inferior device performance should mainly be ascribed to the low Φ_{PL}, less efficient RISC process and unbalanced carrier mobility.

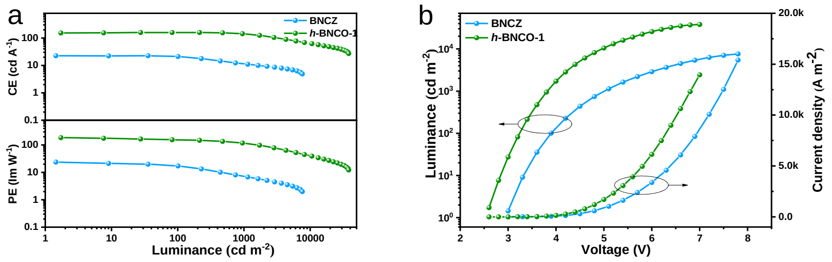

Supplementary Fig. 6. a, CE−luminance and PE−luminance characteristics and **b**, luminance−voltage−current density characteristics of the efficiency-optimized OLEDs.

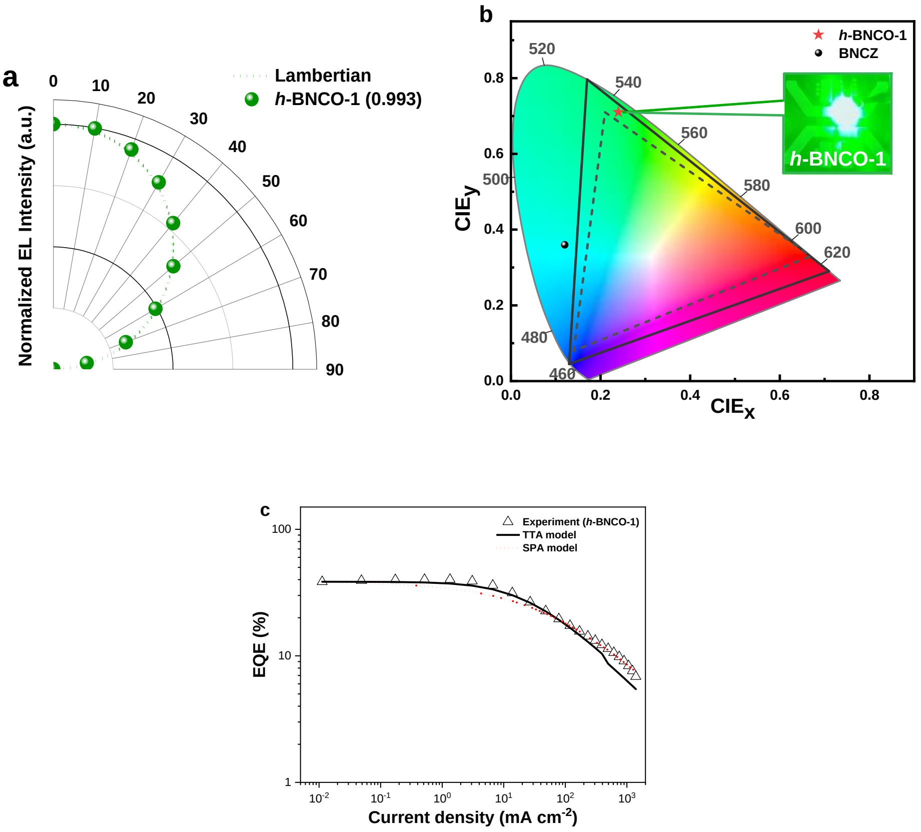

Supplementary Fig. 7. a, Angular-dependent EL emission profile, **b,** CIE coordinates in the CIE 1931 colour space and a photograph that shows the EL emission **c**, EQE versus current density characteristics fitted with TTA and SPA models[1] of the OLED device based on 1 wt% doped *h*-BNCO-1 in DMIC-TRZ.

Supplementary Table 2. Summary of performance of the ever-reported MR-TADF OLEDs.

Framework	Emitter	EML component Nos	λ_{EL}[a] [nm]	FWHM[b] [nm]	k_{RISC}[c] [10^4 s^{-1}]	EQE[d] (%) Max	@1000 cd m^{-2}	Roll-off	CIE[e] (x, y)	Ref
B/N/C=O	*h*-BNCO-1	2	528	39	108	40.1	34.6	14	(0.24, 0.71)	This work
N/C=O	3-PhQAD	2	480	44	1.2	19.1	-	-	(0.13, 0.32)	1
	7- PhQAD	2	472	34	0.64	18.7	-	-	(0.12, 0.24)	1
	QAD-Cz	2	494	57	4.4	20.3	0.73	96	(0.16, 0.47)	2
	QAD-2Cz	2	530	56	8.4	27.3	12.4	55	(0.30, 0.65)	2

	QAD-mTDPA	2	589	67	4.4	26.3	4.7	82	(0.55, 0.44)	2
	mBDPA-TOAT	2	600	45	2.0	17.3	-	-	(0.61, 0.39)	3
	pBDPA-TOAT	2	624	62	1.1	11.3	-	-	(0.66, 0.34)	3
	DiKTa	2	465	37	4.6	14.7	3.3	78	(0.14, 0.18)	4
	Mes$_3$DiKTa	2	480	37	3.1	21.1	4.5	79	(0.12, 0.32)	4
	Cz-DiKTa	2	511	62	-	24.9	13	48	(0.24, 0.61)	5
	Cz-Ph-DiKTa	2	492	61	-	23.0	10.2	56	(0.18, 0.50)	5
	TMCz-DiKTa	2	527	78	-	20.2	16.7	17	(0.32, 0.60)	5
	DMAC-DiKTa	2	549	89	-	23.8	19.9	16	(0.40, 0.57)	5
	3Cz-DiKTa	2	547	54	-	24.4	6.2	75	(0.39, 0.60)	5
	Cz-DiKTa	2	511	62	-	24.9	13	48	(0.24, 0.61)	5
	3TPA-DiKTa	2	551	62	2.49	30.8	7.3	76	(0.41, 0.58)	6
	3DPA-DiKTa	2	613	60	0.14	16.7	1.9	89	(0.63, 0.37)	6
	QAO	2	468	39	-	19.4	-	-	(0.13, 0.18)	7
	QAO-DAd	2	552	-	-	23.9	-	-	(0.41, 0.56)	7
	DQAO	2	472	34	-	15.2	-	-	(0.12, 0.18)	8
	OQAO	2	532	45	-	20.3	-	-	(0.32, 0.65)	8
	SQAO	2	564	72	-	17.8	-	-	(0.47, 0.52)	8
	QA-PF	2	474	27	4.9	16.8	-	-	(0.12, 0.17)	9
	QA-PCN	2	473	30	16.1	16.9	-	-	(0.12, 0.18)	9
	QA-PMO	2	484	27	2.2	15.0	-	-	(0.11, 0.30)	9
	QA-PCZ	2	482	29	5.0	17.5	-	-	(0.11, 0.28)	9
	QA-1	2	455	49	1.5	17.1	-	-	(0.14, 0.12)	10
	QA-2	2	463	37	85	19.0	-	-	(0.13, 0.14)	10
	QA-3	2	515	67	5.3	18.6	-	-	(0.26, 0.62)	10
B/N	DABNA-1	2	459	28	0.99	13.5	-	-	(0.13, 0.09)	11
	DABNA-2	2	467	28	1.48	20.2	-	-	(0.13, 0.14)	11
	PAB	2	456	31	6.95	14.7	-		(0.15, 0.08)	12
	2tPAB	2	456	27	6.34	16.8	-	-	(0.15, 0.08)	12
	3tPAB	2	460	26	5.74	19.3	--	-	(0.14, 0.08)	12
	tDMAC-BN	3	460	28	1.16	21.6	5.4	75	(0.14, 0.09)	13
	tDPAC-BN	3	472	34	4.80	22.3	10.4	53	(0.12, 0.19)	13
	B2	2	460	37	0.93	18.3	-	-	(0.13, 0.11)	14
	ν-DABNA	2	469	18	20	34.4	26.0	24	(0.12, 0.11)	15
	OAB-ABP-1	2	505	33	4	21.8	17.4	20	(0.12, 0.63)	16
	DABNA-NP-TB	2	457	33	1.4	19.5	12.0	38	(0.14, 0.11)	17
	Cz2DABNA-NP-M/TB	2	477	27	7.3	21.8	14.9	32	(0.11, 0.23)	17
	CzB2-M/P	2	497	29	5.1	26.7	18.0	33	(0.12, 0.57)	17
	ν-DABNA-O	2	465	23	16	29.5	26.9	9	(0.13, 0.10)	18
	V-DABNA-Mes	2	480	27	44	22.9	10.9	52	(0.09, 0.21)	19
	V-DABNA	2	483	17	57	26.2	25.3	3	(0.09, 0.27)	20
	V-DABNA-F	2	468	15	65	26.6	23.4	12	(0.12, 0.10)	20
	ν-DABNA-CN-Me	2	504	23	10	31.6	28.5	10	(0.13, 0.65)	21
	ω-DABNA	2	512	25	12.0	31.1	29.4	5	(0.13, 0.73)	22
	DtBuCzB	2	490	31	0.88	26.3	9.0	66	(0.10, 0.45)	23
	TCzBN-DPF	2	498	31	3.45	26.4	12	55	(0.10, 0.56)	23
	TCzBN-TMPh	2	488	27	1.83	25.1	6.5	74	(0.11, 0.38)	23
	TCzBN-oPh	2	492	28	1.62	26.0	10.4	60	(0.09, 0.46)	23
	TCZ-F-DABNA	2	588	61	7.80	39.2	7.84	80	(0.54, 0.44)	24
	DBTN-2	2	520	29	10.7	35.2	20.4	42	(0.19, 0.74)	25
	TW-BN	2	488	26	0.76	27.8	10.7	62	(0.14, 0.36)	26
	TPh-BN	2	492	29	1.43	28.9	15.6	46	(0.10, 0.46)	26
	pCz-BN	2	496	30	0.64	27.2	12.2	55	(0.13, 0.54)	26
	mCz-BN	2	496	31	1.64	25.9	14.0	46	(0.15, 0.55)	26
	Cz-PTZ-BN	2	520	54	8.1	27.6	17.3	37	(0.26, 0.65)	27
	2Cz-PTZ-BN	2	516	56	10.5	32.8	23.5	28	(0.24, 0.63)	27
	2F-BN	3	501	40	2.2	22	15	32	(0.16, 0.60)	28
	3F-BN	3	499	39	3.9	22.7	21.1	7	(0.20, 0.58)	28
	4F-BN	3	493	32	3.9	20.9	16.4	22	(0.12, 0.48)	28
	AZA-BN	3	528	31	0.75	25.7	9	65	(0.28, 0.69)	29
	BN-ICz-1	3	522	24	2.9	24.1	10.6	56	(0.24, 0.73)	30
	BN-ICz-2	3	523	24	6.4	22.2	14.4	35	(0.23, 0.72)	30
	BIC-mCz	3	432	42	4.0	19.4	-	-	(0.16, 0.05)	31
	BIC-pCz	3	466	48	3.1	39.8	-	-	(0.14, 0.16)	31
	mDBIC	3	431	42	5.0	13.5	-	-	(0.16, 0.05)	31
	pDBIC	3	535	30	-	31.0	-	-	(0.33, 0.64)	31
	m-Cz-BNCZ	2	528	45	108	31.4	17.5	44	(0.26, 0.68)	32
	S-Cz-BN	3	488	26	1.8	30.5	26.2	13	(0.12, 0.43)	33
	D-Cz-BN	3	488	24	1.8	37.2	34.3	8	(0.11, 0.43)	33
	R-BN	3	664	48	6.7	28.1	-	-	(0.72, 0.28)	34
	R-TBN	3	686	49	2.5	27.6	-	-	(0.72, 0.28)	34
	C-BN	3	453	25	1.8	26.6	8.9	67	(0.14, 0.07)	35
	m[B-N]N1	3	479	27	1.59	36.0	32.3	10	(0.12, 0.27)	36
	m[B-N]N2	3	485	33	1.44	33.4	29.7	11	(0.11, 0.32)	36

**DtBuPhCzB**	**2**	**504**	**34**	**-**	**23.4**	**5.7**	**76**	**(0.15, 0.61)**	**37**
**(R)-OBN-2CN-BN**	**2**	**496**	**30**	**0.95**	**29.4**	**8.1**	**72**	**(0.11, 0.52)**	**38**
**(R)-OBN-4CN-BN**	**2**	**508**	**33**	**0.83**	**24.5**	**2.1**	**91**	**(0.14, 0.64)**	**38**
**DtCzB-DPTRZ**	**3**	**532**	**39**	**0.1**	**24.6**	**4.7**	**81**	**(0.33, 0.63)**	**39**
**DtCzB-CNPm**	**3**	**540**	**44**	**0.14**	**25**	**5.7**	**77**	**(0.35, 0.63)**	**39**
**DtCzB-TPTRZ**	**3**	**516**	**38**	**1.08**	**29.8**	**12.4**	**58**	**(0.18, 0.67)**	**39**
**DtCzB-PPm**	**3**	**508**	**33**	**1.02**	**28.6**	**10.2**	**64**	**(0.16, 0.66)**	**39**
**BN-TP**	**2**	**528**	**36**	**2.09**	**35.1**	**20.8**	**41**	**(0.26, 0.70)**	**40**
**DBNO**	**3**	**504**	**24**	**3**	**35.9**	**5.9**	**84**	**(0.18, 0.60)**	**41**
***m*-PCz-BNCZ**	**2**	**504**	**29**	**1.29**	**36.1**	**18.6**	**48**	**(0.11, 0.61)**	**42**
***m*-DPAcP–BNCZ**	**2**	**496**	**28**	**1.16**	**42.0**	**17**	**60**	**(0.09, 0.54)**	**42**
***m*-BN–BNCZ**	**2**	**492**	**28**	**0.93**	**35.0**	**10.9**	**69**	**(0.09, 0.48)**	**42**
***m*-SF–BNCZ**	**2**	**496**	**28**	**1.16**	**41.1**	**17.9**	**56**	**(0.09, 0.53)**	**42**
**BN-R**	**3**	**617**	**47**	**1.1**	**22**	**7.1**	**68**	**(0.65, 0.34)**	**43**
**Tip-DtCzB**	**2**	**484**	**25**	**0.92**	**28.9**	**6.8**	**76**	**(0.10, 0.29)**	**44**
***t*DPA-DtCzB**	**2**	**480**	**27**	**2.45**	**25.0**	**8.3**	**67**	**(0.11, 0.23)**	**44**
***t*-DABNA**	**3**	**464**	**26**	**1.17**	**28.4**	**4.4**	**85**	**(0.13, 0.10)**	**45**
***t*-DAB-DPA**	**3**	**459**	**26**	**3.97**	**27.9**	**8.1**	**71**	**(0.13, 0.08)**	**45**
***t*-DABNA-dtB**	**3**	**471**	**23**	**2.08**	**11.3**	**10.9**	**4**	**(0.13, 0.08)**	**46**
**B-dpa-Cz**	**3**	**475**	**27**	**2.74**	**20.1**	**4.7**	**77**	**(0.11, 0.19)**	**47**
**B-dpa-dmAc**	**3**	**478**	**32**	**4.48**	**24.3**	**14.9**	**39**	**(0.11, 0.22)**	**47**
**B-dpa-SpiroAc**	**3**	**481**	**33**	**4.97**	**25.1**	**15.3**	**39**	**(0.10, 0.27)**	**47**
**BN1**	**2**	**506**	**36**	**1.9**	**24.3**	**12.9**	**47**	**(0.15, 0.63)**	**48**
**BN2**	**2**	**545**	**46**	**1.5**	**24.5**	**7.6**	**69**	**(0.38, 0.61)**	**48**
**BN3**	**2**	**568**	**43**	**1.4**	**24.7**	**8.9**	**64**	**(0.47, 0.52)**	**48**
**DPACzBN1**	**2**	**475**	**34**	**1.2**	**23.6**	**9.6**	**59**	**(0.14, 0.30)**	**49**
**DPACzBN2**	**2**	**469**	**28**	**2.9**	**24.0**	**14.3**	**40**	**(0.13, 0.16)**	**49**
**DPACzBN3**	**2**	**472**	**24**	**2.1**	**27.7**	**6.7**	**76**	**(0.12, 0.18)**	**49**
**BN-DMAC**	**2**	**502**	**48**	**9.6**	**21.1**	**12.5**	**41**	**(0.14, 0.54)**	**50**
**BN-DPAC**	**2**	**516**	**50**	**12.6**	**23.5**	**16.3**	**31**	**(0.21, 0.65)**	**50**
**R-CzOBN**	**3**	**510**	**42**	**1.4**	**33.2**	**13.4**	**60**	**(0.21, 0.66)**	**51**
**S-CzOBN**	**3**	**510**	**42**	**1.4**	**32.9**	**13.3**	**60**	**(0.21, 0.66)**	**51**
**BN-CP1**	**2**	**496**	**25**	**3.12**	**40.0**	**18.5**	**54**	**(0.09, 0.50)**	**52**
**BN-CP2**	**2**	**499**	**30**	**3.52**	**30.4**	**13.3**	**56**	**(0.13, 0.59)**	**52**
**(*R*)-BN-MeIAc**	**2**	**504**	**33**	**6.3**	**37.2**	**26.1**	**30**	**(0.12, 0.63)**	**53**
**(*S*)-BN-MeIAc**	**2**	**503**	**33**	**6.3**	**36.1**	**25.1**	**30**	**(0.12, 0.62)**	**53**
**BN1**	**3**	**457**	**28**	**1.3**	**31.2**	**9.3**	**70**	**(0.14, 0.08)**	**54**
**BN2**	**3**	**467**	**23**	**2.6**	**32.2**	**15.5**	**52**	**(0.13, 0.11)**	**54**
**BN3**	**3**	**458**	**23**	**25.5**	**37.6**	**26.2**	**30**	**(0.14, 0.08)**	**54**
**DMAc-BN**	**2**	**503**	**49**	**2.4**	**20.3**	**12.0**	**41**	**(0.18, 0.60)**	**55**
**PXZ-BN**	**2**	**516**	**47**	**0.9**	**23.3**	**11.3**	**52**	**(0.22, 0.67)**	**55**
**BN2**	**3**	**547**	**-**	**21**	**19.9**	**13.5**	**32**	**(0.16, 0.57)**	**56**
**TCz-BN2**	**3**	**554**	**-**	**24.4**	**25.1**	**18.7**	**25**	**(0.41, 0.56)**	**56**
**mICz-DABNA**	**2**	**466**	**26**	**2.65**	**26.4**	**-**	**-**	**(0.13, 0.11)**	**57**
**BFCz-DABNA**	**2**	**463**	**26**	**2.78**	**28.0**	**-**	**-**	**(0.13, 0.09)**	**57**
**m-*v*-DABNA**	**2**	**471**	**18**	**23.0**	**36.2**	**-**	**-**	**(0.12, 0.12)**	**58**
**4F-*v*-DABNA**	**2**	**464**	**18**	**22.8**	**35.8**	**-**	**-**	**(0.13, 0.08)**	**58**
**4F-m-*v*-DABNA**	**2**	**461**	**18**	**21.0**	**33.7**	**-**	**-**	**(0.13, 0.06)**	**58**
**BBCZ-DB**	**2**	**469**	**27**	**1.9**	**29.3**	**-**	**-**	**(0.12, 0.18)**	**59**
**BBCZ-SB**	**2**	**487**	**26**	**1.4**	**27.8**	**-**	**-**	**(0.41, 0.56)**	**59**
**BBCZ-R**	**2**	**515**	**54**	**18**	**31.8**	**-**	**-**	**(0.26, 0.68)**	**59**
**BBCZ-G**	**2**	**549**	**48**	**10**	**29.3**	**-**	**-**	**(0.41, 0.56)**	**59**
**BBCZ-Y**	**2**	**616**	**26**	**1.2**	**22.0**	**-**	**-**	**(0.67, 0.33)**	**59**
**γ-Cb-B**	**2**	**461**	**28**	**5.8**	**19.0**	**7.7**	**59**	**(0.13, 0.13)**	**60**
**Cz-B**	**2**	**482**	**30**	**2.7**	**22.6**	**6.9**	**69**	**(0.11, 0.31)**	**60**
**TCz-B**	**2**	**515**	**30**	**1.3**	**29.2**	**9.4**	**68**	**(0.16, 0.71)**	**60**
**DACz-B**	**2**	**571**	**44**	**1.0**	**19.6**	**4.8**	**76**	**(0.47, 0.51)**	**60**
**pBP-DABNA-Me**	**3**	**464**	**23**	**6.85**	**22.4**	**-**	**-**	**(0.13, 0.09)**	**61**
**mBP-DABNA-Me**	**3**	**468**	**28**	**1.95**	**24.3**	**-**	**-**	**(0.12, 0.14)**	**62**
**(+)-BN4**	**2**	**510**	**49**	**16**	**20.6**	**10.7**	**48**	**(0.19, 0.63)**	**63**
**(-)-BN4**	**2**	**512**	**49**	**3.7**	**19**	**10.1**	**47**	**(0.21, 0.64)**	**63**

	(+)-BN5	2	506	48	7.4	22	10.9	50	(0.17, 0.59)	63
	(-)-BN5	2	506	48	3.3	26.5	11.1	58	(0.17, 0.60)	63
	TPXZBN	2	506	37	4.8	21.3	17.4	18	(0.16, 0.65)	64
	DPXZCZBN	2	505	36	11.1	19.2	17.2	10	(0.15, 0.64)	64
	DBON	2	510	29	8	26.7	12	55	(0.17, 0.68)	65
	SF1BN	2	492	28	9.12	35.9	14.0	61	(0.08, 0.47)	66
	SF3BN	2	496	30	3.31	32.2	10.3	68	(0.09, 0.52)	66
	CNCz-BNCZ	3	583	49	42	33.7	16.4	51	(0.54, 0.46)	67
	TRZCzPh-BNCZ	2	513	37	88	32.5	22.9	30	(0.17, 0.68)	68
	TRZTPh-BNCZ	2	513	33	75	31.4	23.1	26	(0.16, 0.70)	68
	NBNP	2	502	33	30	28	22.4	20	(0.12, 0.62)	69
	tCzphB-Fl	2	535	26	-	26.2	-	-	(0.26, 0.72)	70
	tCzphB-Ph	2	527	24	-	29.3	-	-	(0.21, 0.75)	70
	NOBNacene	2	412	41	0.37	11.2	-	-	(0.18, 0.07)	71
	D-VTCzBN	2	499	38	100	31.7	19.8	38	(0.14, 0.56)	72
	D-TCz-VTCzBN	2	524	37	90	32.2	16	50	(0.22, 0.71)	72
	B-O-dpa	2	443	32	0.83	16.3	--	-	(0.15, 0.05)	73
	B-O-Cz	2	481	63	4.02	20.3	-	-	(0.13, 0.22)	73
	B-O-dmAc	2	475	44	1.82	23.1	-	-	(0.12, 0.21)	73
	B-O-dpAc	2	473	42	3.12	22.8	-	-	(0.12, 0.20)	73
Heavy atom effect	BNCZPXZ	2	520	49	2.31	25.7	10.4	60	(0.25, 0.67)	74
	BNCZPXZ	2	524	57	15.4	28.7	20.7	28	(0.27, 0.65)	74
	(SIPr)AuBN	3	511	40	-	24.8	24.3	2	(0.20, 0.69)	75
	(BzIPr)AuBN	3	510	34	500	30.3	28.1	7	(0.16, 0.68)	75
	(PyIPr)AuBN	3	512	37	320	27.6	20.5	26	(0.18, 0.69)	75
	(PzIPr)AuBN	3	515	39	-	24	23.1	4	(0.22, 0.67)	75
	TBN-TPA	2	474	27	-	32.1	13.9	57	(0.12, 0.19)	76
	CzBNO	2	454	36	3.47	14.7	4.0	73	(0.14, 0.08)	77
	DMAcBNO	2	472	41	1.40	31.2	11.6	63	(0.13, 0.19)	77
	DPAcBNO	2	468	37	1.75	28.0	9.8	65	(0.13, 0.14)	77
	PTZBN1	2	496	47	11.1	26.9	17.9	33	(0.16, 0.51)	78
	PTZBN2	2	483	43	45.1	30.5	23.0	25	(0.13, 0.31)	78
	PTZBN3	2	476	36	10.8	19.9	12.1	39	(0.13, 0.22)	78
	BSBS-N1	2	478	25	190	27.8	-	-	(0.11, 0.22)	79
	BOBO-Z	2	445	18	7	13.6	3.3	76	(0.15, 0.04)	80
	BOBS-Z	2	456	23	86	26.9	15.0	44	(0.14, 0.06)	80
	BSBS-Z	2	463	22	160	26.8	15.9	41	(0.13, 0.08)	80
	CzBO	2	448	30	0.9	13.4	3.5	74	(0.15, 0.05)	81
	CzBS	2	473	31	22	23.1	15.0	35	(0.11, 0.16)	81
	CzBSe	2	481	33	18000	23.9	20.0	16	(0.10, 0.24)	81
	Cz-BSN	2	482	32	9.6	18.9	6.8	64	(0.11, 0.28)	82
	DCz-BSN	2	473	29	10.4	22.0	10.0	55	(0.11, 0.17)	82
	Cz-BSeN	2	490	36	750	20.3	-	-	(0.13, 0.45)	83
	DCz-BSeN	2	481	32	880	22.3	-	-	(0.11, 0.25)	83
	2PXZBN	3	522	60	1.03	17.7	7.4	58	(0.28, 0.64)	84
	2PTZBN	3	528	58	2.76	25.5	17.2	33	(0.28, 0.65)	84
	2PXZBN	2	517	49	4.3	30.7	17.9	42	(0.23, 0.67)	85
	2PTZBN	2	520	52	19	34.6	29.5	15	(0.24, 0.67)	85
	BNSSe	2	520	50	60	35.7	32	10	(0.22, 0.66)	85
	BNSeSe	2	514	48	200	36.8	34	8	(0.19, 0.66)	85
	DCzBN-Au	2	508	34	2300	35.78	35.76	0.06	(0.16, 0.67)	86
	(*P*)-helicene-BN	2	523	49	4.6	31.5	18.7	41	(0.26, 0.66)	87
	(*M*)-helicene-BN	2	524	50	4.6	30.7	17.9	42	(0.26, 0.66)	87
	BN-STO	2	517	34	12	40.1	28.1	30	(0.19, 0.70)	88

[a] Peak wavelength of the EL spectrum. [b] Full-width at half-maximum. [c] The rate constant of reverse intersystem crossing. [d] External quantum efficiency of maximum, external quantum efficiency at a luminance of 1000 cd m^{-2}, and efficiency roll-offs (at 1000 cd m^{-2}). [e] CIE coordinates with EQE_{max}.

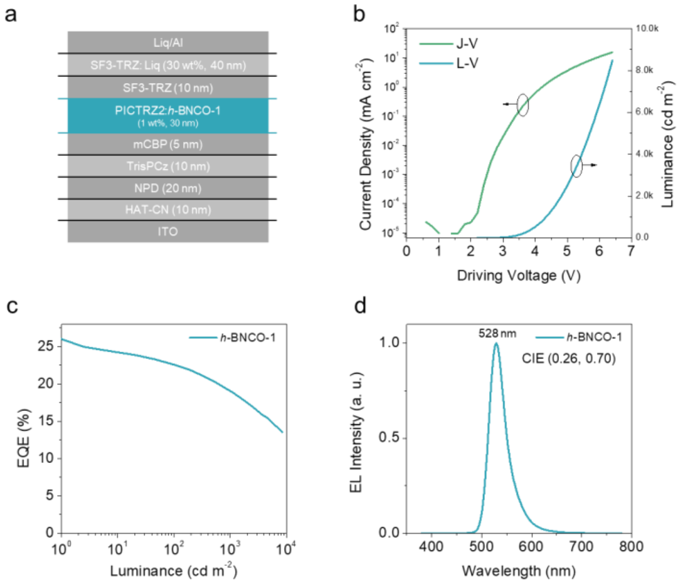

Supplementary Fig. 8. High-stability OLED performance based on *h*-BNCO-1. **a**, Device structure. **b**, Current density−driving voltage−luminance (J−V−L) characteristics. **c**, EQE-to-luminance curve. **d**, EL spectrum at 1000 cd m^{-2}.

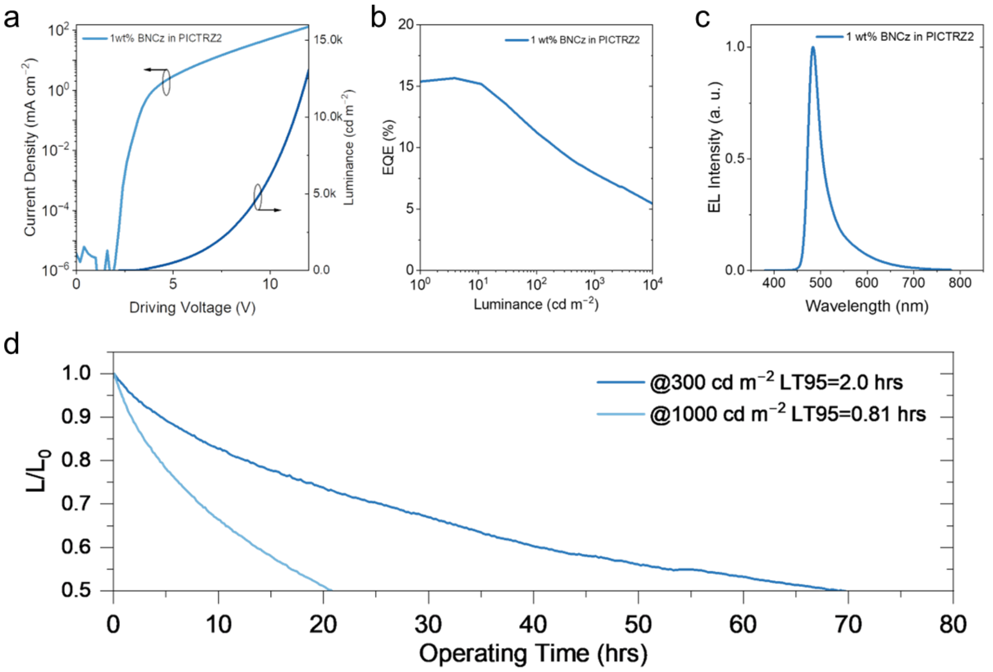

Supplementary Fig. 9. Performance of the control device based on BNCZ with the same device structure as the high-stability *h*-BNCO-1 based device. (a) Current density-driving voltage-luminance curve, (b) EQE versus luminance curve, (c) EL spectrum, and (d) device operation lifetime at the initial luminance of 300 and 1000 cd m^{-2}, respectively.

The maximum EQE of BNCz-based OLED was around 15.7%. However, the efficiency roll-off (~50% at 1000 cd m^{-2}) was much larger than that of *h*-BNCO-1-based device. This can be ascribed to the triplet-caused annihilations due to the slower RISC process. Resultantly, the LT_{95} for BNCz-based device lifetime was only 0.81 h (initial luminance of 1000 cd m^{-2}) and 2.0 h (initial luminance of 300 cd m^{-2}). It was worth noting that LT_{95} for *h*-BNCO-1-based device was over 100 times longer than that of BNCz.

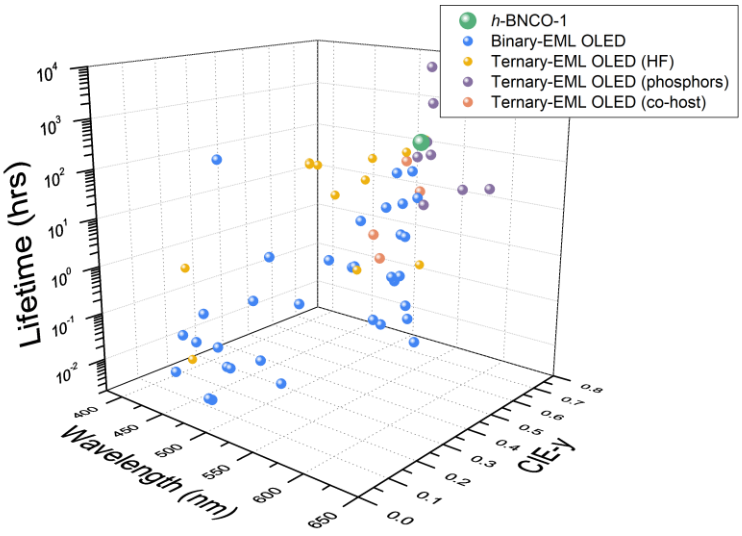

Supplementary Fig. 10. A summary of the operational lifetime (LT_{95})-wavelength peak-CIE-y of the reported MR-OLEDs based on binary-EMLs, ternary-EMLs with assistance of HF, phosphors, and co-host system.

Supplementary Table 3. Summary of the reported OLED device lifetime based on MR-TADF emitters.

	EML component Nos	λ_{EL} (nm)	FWHM (nm/eV)	CIE (x, y)	LT_{95} (hrs)	LT_{90} (hrs)	LT_{80} (hrs)	LT_{50} (hrs)
This work	2	528	38/0.17	(0.26,0.70)	137	/	/	/
Ref. 28 2F-BN	3 (HF)	501	40/-	(0.16,0.60)	~20[d] ~67[b,c]	45.76[d] 153.9[b,c]	/	/
Ref. 28 3F-BN	3 (HF)	499	38.5/-	(0.20,0.58)	~7[d] ~24[b,c]	15.53[d] 52.2[b,c]	/	/
Ref. 28 4F-BN	3 (HF)	493	31.6/-	(0.12,0.48)	~4.5[d] ~15[b,c]	10.35[d] 34.8[b,c]	/	/
Ref. 29 AZA-BN	2	527	30/-	(0.27,0.69)	~2.5[d] ~8.4[b,c]	~5[d] ~16.8[b,c]	/	/
Ref. 29 AZA-BN	3 (HF sensitized by phosphor)	527	30/-	(0.27,0.69)	~20[d] ~67.3[b,c]	46.3[d] ~155.7[b,c]	/	/
Ref. 49 DPACzBN2	2	469	28/-	(0.13,0.16)	/	/	/	~9[e] ~0.16[b,c]
Ref. 16 OAB-ABP-1	2	505	33/-	(0.12,0.63)	/	/	/	11[f] 1.3[b,c]

Ref. 18<br>υ-DABNA-O-Me	2	465	23/-	(0.13,0.10)	/	~20[e]<br>~0.36[b,c]	/	314[e]<br>~5.6[b,c]
Ref. 50<br>BN-DMAC	3<br>(co-host)	506	56/0.27	(0.19,0.59)	~5[g]<br>~1.5[b,c]	~18[g]<br>~5.4[b,c]	82[g]<br>~24.4[b,c]	/
Ref. 50<br>BN-DPAC	3<br>(co-host)	508	49/0.23	(0.16,0.61)	~1.2[g]<br>~0.4[b,c]	~2.8[g]<br>~0.8[b,c]	8[g]<br>~2.4[b,c]	/
Ref. 17<br>CzB2-M/TB	2	497	29	(0.12,0.57)	~10[g]<br>~3.0[b,c]	~28[g]<br>~8.3[b,c]	74[g]<br>~22[b,c]	/
Ref. 17<br>Cz2DABNA-NP-M/TB	2	477	27	(0.11,0.23)	~8[e]<br>~0.2[b,c]	~32[e]<br>~0.6[b,c]	91[e]<br>~1.6[b,c]	/
Ref. 17<br>DABNA-NP-TB	2	477	33	(0.14,0.11)	~2[e]<br>~0.04[b,c]	~8[e]<br>~0.14[b,c]	19[e]<br>~0.34[b,c]	/
Ref. 60<br>TCz-B	2	515	30/0.14	(0.16,0.71)	/	/	/	2[e]<br>~0.04[b,c]
Ref. 60<br>DACz-B	2	571	44/0.17	(0.47,0.51)	~1[e]<br>~0.02[b,c]	~2[e]<br>~0.04[b,c]	/	10.9[e]<br>~0.2[b,c]
Ref. 60<br>Cz-B	2	482	30/0.16	(0.11,0.31)	/	/	/	1.5[e]<br>~0.03[b,c]
Ref. 60<br>γ-Cb-B	2	461	28/0.16	(0.13,0.13)	/	/	/	0.9[e]<br>~0.02[b,c]
Ref. 20<br>V-DABNA-F	2	468	15/0.087	(0.12,0.10)	/	/	/	3.6[g]<br>~1.1[b,c]
Ref. 20<br>V-DABNA	2	483	17/0.092	(0.09,0.27)	~5[g]<br>~1.5[b,c]	~10[g]<br>~3.0[b,c]	/	184[g]<br>~54.7[b,c]
Ref. 86<br>DCzBN-Au	2	510	34/0.16	(0.16,0.67)	27.5[c]	73.4[c]	150.1[c]	/
Ref. 21<br>υ-DABNA-CN-Me	2	504	23/0.112	(0.13,0.65)	~7[h]<br>~4.7[b,c]	~18[h]<br>~12.2[b,c]	59[h]<br>~39.9[b,c]	/
Ref. 48<br>t-DABNA-dTB	2	471	23/-	(0.117,0.139)	208[c]	/	/	/
Ref. 48<br>t-DABNA-dTB	2 (tandem)	471	23/-	(0.119,0.115)	502[c]	/	/	/
Ref. 80<br>BSBS-Z	2	463	21/0.12	(0.13,0.08)	/	5[e]<br>~0.09[b,c]	/	37.4[e]<br>~0.67[b,c]
Ref. 80<br>BOBS-Z	2	455	23/0.12	(0.14,0.06)	/	8[e]<br>~0.14[b,c]	/	34[e]<br>~0.60[b,c]
Ref. 80<br>BOBO-Z	2	445	19/0.11	(0.16,0.06)	/	<1[e]<br><0.02[b,c]	/	4.4[e]<br>~0.08[b,c]
Ref. 68<br>TRZCzPh-BNCz	2	513	37/-	(0.17,0.68)	~3.5[g]<br>~1.1[b,c]	~7[g]<br>~2.1[b,c]	/	14.7[g]<br>~4.4[b,c]
Ref. 68<br>TRZTPh-BNCz	2	513	30/-	(0.17,0.70)	~3 [g]<br>~0.9[b,c]	~7[g]<br>~2.1[b,c]	/	13.4[g]<br>~4.0[b,c]
Ref. 68<br>DtCzB-DPTRZ	2	532	40/-	(0.32,0.63)	~0.1 [g]<br>~0.04[b,c]	~0.2[e]<br>~0.07[b,c]	/	0.6[g]<br>~0.2[b,c]
Ref. 75<br>(BzIPr)AuBN	3<br>(co-host)	513	34/-	(0.18,0.70)	47.2[c]	~0.3[i]<br>~129[c]	/	/
Ref. 27<br>Cz-PTZ-BN	2	520	54/-	(0.26,0.65)	<0.5[g]<br><0.15[b,c]	<1[g]<br><0.3[b,c]	/	14.1[g]<br>~4.2[b,c]
Ref. 27<br>2Cz-PTZ-BN	2	516	56/-	(0.26,0.63)	<0.5[g]<br><0.15[b,c]	<1[g]<br><0.3[b,c]	/	10.3[g]<br>~3.1[b,c]
Ref. 54<br>BN3	3 (HF)	458	23/0.131	(0.14,0.08)	~1.25[e]<br>~0.02[b,c]	~2.5[e]<br>~0.04[b,c]	/	10.3[e]<br>~0.18[b,c]
Ref. 81<br>CzBSe	2	481	33/0.18	(0.10,0.24)	<0.5[e]<br><0.01[b,c]	<0.5[e]<br><0.01[b,c]	/	7.48[e]<br>~0.13[b,c]
Ref. 81<br>CzBS	2	473	31/0.17	(0.11,0.16)	<0.5[e]<br><0.01[b,c]	<0.5[e]<br><0.01[b,c]	/	4.23[e]<br>~0.08[b,c]
Ref. 85<br>BNSeSe	2	512	48/-	(0.19,0.66)	<0.1[c]	<0.3[c]	/	4.1[c]
Ref. 85<br>2PXZBN	2	517	49/-	(0.23,0.67)	~6[c]	~17[c]	/	158[c]
Ref. 85<br>BN3	3 (HF)	558	40/-	(0.41,0.58)	<0.6[c]	<2[c]	/	51.1[c]
Ref. 88<br>BN-STO	2	517	34/0.16	(0.19,0.70)	~1[c]	~2[c]	/	35[c]

Ref. 88<br>BN-XTO	2	516	34/0.16	(0.19,0.70)	~25[c]	~36[c]	/	354[c]
Ref. 26<br>TM-BN	2	488	26/0.13	(0.14,0.36)	0.3[g]<br>~0.1[b,c]	0.7[g]<br>~0.2[b,c]	/	10.4[g]<br>~3.1[b,c]
Ref. 26<br>TPh-BN	2	492	28/0.14	(0.10,0.46)	2[g]<br>~0.6[b,c]	~4[g]<br>~1.2[b,c]	/	36.5[g]<br>~10.9[b,c]
Ref. 26<br>pCz-BN	2	496	30/0.15	(0.13,0.54)	<1[g]<br>~0.3[b,c]	<2[g]<br><0.6[b,c]	/	27.3[g]<br>~8.1[b,c]
Ref. 26<br>mCz-BN	2	496	31/0.15	(0.15,0.55)	<1[g]<br>~0.3[b,c]	<2[g]<br><0.6[b,c]	/	18.6[g]<br>~5.5[b,c]
Ref. 40<br>BN-TP	3<br>(co-host)	528	36/-	(0.26,0.70)	<1[j]<br><11.3[b,c]	~1[j]<br>~11.3[b,c]	/	28.8[j]<br>~326[b,c]
Ref. 43<br>BN-R	2	617	47/0.15	(0.654,0.344)	/	/	/	94.7[k]<br>~75.6[b,c]
Ref. 43<br>BN-R	3<br>(HF sensitized by phosphor)	618	47/0.15	(0.663,0.337)	<20[l]<br>~52.2[b,c]	~50[l]<br>~130.6[b,c]	/	416.7[l]<br>~1089[b,c]
Ref. 22<br>ω-DABNA	2	512	25/0.114	(0.13,0.73)	25[c]	~80[c]	292[c]	/
Ref. 34<br>R-BN	3<br>(HF sensitized by phosphor)	664	48/-	(0.719,0.280)	~55[d]<br>~185[b,c]	125[d]<br>~420[b,c]		
Ref. 34<br>R-BN	3<br>(HF sensitized by phosphor)	686	49/-	(0.721,0.278)	~70[d]<br>~235[b,c]	~151[d]<br>~508[b,c]		
Ref. 35<br>C-BN	3 (HF)	453	28/-	(0.14,0.08)	~5[g]<br>~1.5[b,c]	12.3[g]<br>~3.7[b,c]		
Ref. 41<br>DBNO	2	504	24/-	(0.18,0.60)	<0.1[g]<br>~0.03[b,c]	<0.1[g]<br>~0.03[b,c]		<0.5[g]<br>~0.15[b,c]
Ref. 41<br>DBNO	3 (HF)	504	27/-	(0.14,0.53)	<1 [g]<br><0.3[b,c]	<1.5[g]<br><0.5[b,c]		~3[g]<br>~0.9[b,c]
Ref. 30<br>BN-ICz-1	3 (HF)	523	23/0.09	(0.22,0.74)	~40[d]<br>~135[b,c]	82.3[d]<br>277[b,c]		
Ref. 30<br>BN-ICz-2	3 (HF)	523	23/0.09	(0.23,0.73)	~35[d]<br>~118[b,c]	71.3[d]<br>240[b,c]		
Ref. 33<br>DtBuCzB	3 (HF)	488	29/-	(0.13,0.40)	~27[d]<br>~91[b,c]	60.2[d]<br>~202[b,c]		
Ref. 33<br>S-Cz-BN	3 (HF)	488	26/-	(0.12,0.43)	~22[d]<br>~74[b,c]	52.1[d]<br>~175[b,c]		
Ref. 33<br>D-Cz-BN	3 (HF)	488	24/-	(0.11,0.40)	~25[d]<br>~84[b,c]	58.5[d]<br>~197[b,c]		
Ref. 70<br>tCzphB-Ph	3<br>(HF sensitized by phosphor)	527	24/-	(0.21,0.75)	~0.25[m]<br>~61.8[b,c]	0.5[m]<br>~123.6[b,c]		
Ref. 70<br>tCzphB-Fl	3<br>(HF sensitized by phosphor)	535	26/-	(0.26,0.72)	~3.5[n]<br>~944[b,c]	12[n]<br>~3238[b,c]		
Ref. 70<br>tCzphB-Ph	3<br>(HF sensitized by phosphor)	527	-/-	(0.20,0.73)	~0.6[o]<br>~128[b,c]	1.4[o]<br>~298[b,c]		
Ref. 70<br>tCzphB-Fl	3<br>(HF sensitized by phosphor)	535	-/-	(0.26,0.71)	~22[p]<br>~5725[b,c]	70.5[p]<br>~18347[b,c]		

[a] HF indicates hyperfluorescence; [b] LT95/LT90/LT80/LT50 measured at the diverse initial luminance were estimated by using the formula $LT(L_x)=LT(L_0)\times(L_x / L_0)^n$, where L_0 denotes the original luminance, L_x denotes the desired luminance, and n is the lifetime acceleration factor. (For the better comparison, here n was consistent as 1.75); [c] Estimated at luminance of 1000 cd m^{-2}; [d] Estimated at luminance of 2000 cd m^{-2}; [e] Estimated at luminance of 100 cd m^{-2}; [f] Estimated at luminance of 300 cd m^{-2}; [g] Estimated at luminance of 500 cd m^{-2}; [h] Estimated at luminance of 800 cd m^{-2}; [i] Estimated at luminance of 29000 cd m^{-2}; [j] Estimated at luminance of 4000 cd m^{-2}; [k] Estimated at luminance of 876 cd m^{-2}; [l] Estimated at luminance of 1759 cd m^{-2}; [m] Estimated at luminance of 23300 cd m^{-2}; [n] Estimated at luminance of 24500 cd m^{-2}; [o] Estimated at luminance of 21400 cd m^{-2}; [p] Estimated at luminance of 24000 cd m^{-2}.

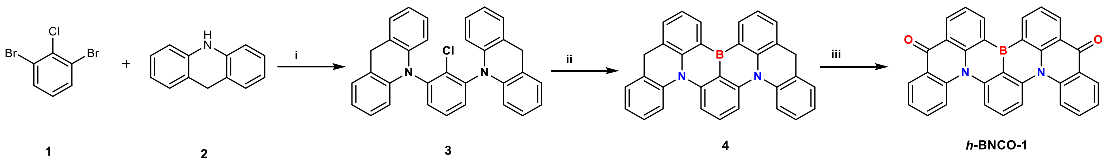

Supplementary Fig. 11. Synthesis route of MR-TADF emitter *h*-BNCO-1.

Reaction conditions: (i) $Pd_2(dba)_3$, *t*BuONa, *t*Bu_3P, toluene; (ii) ①*t*-BuLi, *t*BuPh, ②BBr_3, ③DIEA; (iii) DDQ, DMSO, O_2. The known compounds of BNCZ and PhCzBCz were synthesized and purified according to published procedures.[39,60]

Synthesis of 3 (10,10'-(2-chloro-1,3-phenylene)bis(9,10-dihydroacridine)).

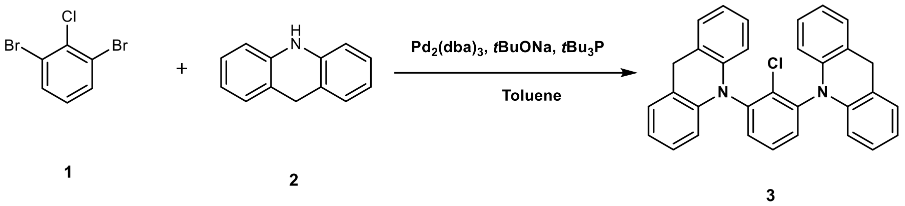

Compound **1** (594 mg, 2.20 mmol), **2** (1.01 g, 5.60 mmol), $Pd_2(dba)_3$ (322 mg, 0.56 mmol), *t*BuONa (2.15 g, 22.4 mmol) and tri-*tert*-butylphosphine (1.00 mL, 1.68 mmol) were added to toluene (30 mL) in a N_2 atmosphere. The mixture was stirred and heated to 110°C for 24 h. After the reaction liquid was cooled to room temperature, the reaction mixture was filtered with a pad of silica gel column and concentrated in vacuo. The crude product was purified by column chromatography on a silica gel column with petroleum ether (PE) as the eluent to obtain **3** (265 mg, 26%). ^{1}H NMR (400 MHz, CD_2Cl_2) δ 7.73 (dd, *J* = 8.5, 7.1 Hz, 1H), 7.64 – 7.60 (m, 2H), 7.17 (dq, *J* = 7.4, 1.2 Hz, 4H), 7.06 – 6.99 (m, 4H), 6.90 (td, *J* = 7.4, 1.2 Hz, 4H), 6.23 (dd, *J* = 8.2, 1.2 Hz, 4H), 4.26 (s, 4H). ^{13}C NMR (101 MHz, CD_2Cl_2) δ 140.83, 140.74, 138.06, 133.79, 130.59, 128.74, 126.90, 121.06, 121.03, 113.19, 31.43. HRMS (EI, *m*/*z*). Calcd for $C_{32}H_{23}ClN_2$: 470.1550; Found: 470.1544.

Synthesis of 4.

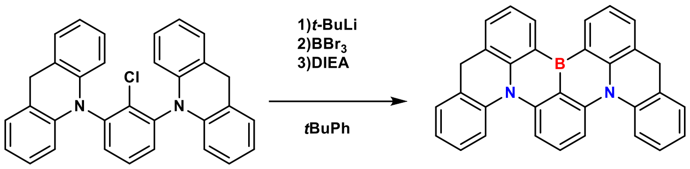

3

4

Under a nitrogen atmosphere, a solution of *t*BuLi (1.6 mol in pentane, 15.6 mL, 25.0 mmol) was slowly added to a solution of compound **3** (4.71 g, 10.0 mmol) in *tert*-butylbenzene (50 mL) at -50 °C. After stirring at 60 °C for 6 h, BBr_3 (2.40 mL, 25.0 mmol) was added dropwise at -50 °C. The mixture was slowly warmed to room temperature and stirred for an additional 6 h. Then, N,N-diisopropylethylamine (0.8 mL, 5 mmol) was added at 0 °C. Finally, the mixture was heated to 130 °C for an additional 12 h. After cooling to room temperature, the reaction mixture was carefully quenched by H_2O, and the pH of the solution was adjusted to 7 by phosphate buffer and concentrated in *vacuo*. The crude product was purified by column chromatography on a silica gel column with petroleum ether (PE): dichloromethane (DCM) = 50:1 as the eluent to obtain **4**. Yield: 530 mg, 12%. ^{1}H NMR (400 MHz, $CDCl_3$) δ 8.38 (dt, *J* = 7.5, 1.3 Hz, 2H), 7.68 – 7.62 (m, 4H), 7.58 (dd, *J* = 9.2, 7.0 Hz, 1H), 7.53 (dt, *J* = 7.3, 1.4 Hz, 2H), 7.39 (ddd, *J* = 5.2, 3.6, 1.0 Hz, 2H), 7.35 (t, *J* = 7.4 Hz, 2H), 7.17 – 7.12 (m, 4H), 4.20 (d, *J* = 16.4 Hz, 2H), 4.03 (d, *J* = 16.5 Hz, 2H). ^{13}C NMR (101 MHz, $CDCl_3$) δ 144.86, 143.40, 141.28, 132.64, 130.94, 130.01, 129.85, 128.08, 126.62, 126.29, 124.44, 122.61, 119.43, 110.36, 77.34, 77.03, 76.71, 33.94, 0.01. HRMS (EI, *m*/*z*). Calcd for $C_{32}H_{21}BN_2$: 444.1798, Found: 444.1794.

Synthesis of *h*-BNCO-1.

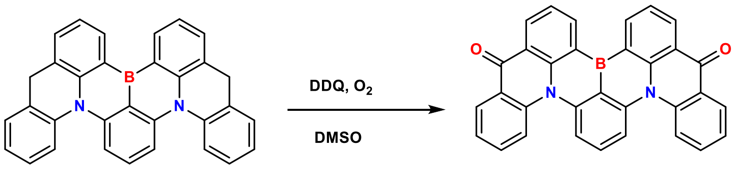

4

h-BNCO-1

Under an oxygen atmosphere, **4** (62 mg, 0.12 mmol) was dissolved in a mixed solution of DMSO (10 mL). 2,3-Dichloro-5,6-dicyanobenzoquinone (810 mg, 3.60 mmol) was slowly added to the reaction flask at room temperature. Finally, the mixture was reacted

at room temperature for an additional 72 h. The reaction mixture was carefully quenched by H_2O and MeOH, and then the pH of the solution was adjusted to 7.0 by phosphate buffer. The organic layer was extracted by DCM 3 times and concentrated in *vacuo*. The crude product was purified by column chromatography on a silica gel column with petroleum ether (PE) as the eluent to obtain ***h*-BNCO-1**. Yield: 5 mg, 2%. ^{1}H NMR (400 MHz, CD_2Cl_2: $CDCl_3$ = 2: 1) δ 8.71 (d, J = 7.7 Hz, 2H), 8.58 (d, J = 7.2 Hz, 2H), 8.46 (d, J = 7.9 Hz, 2H), 8.09 (d, J = 8.4 Hz, 2H), 7.85 (d, J = 8.0 Hz, 2H), 7.64 (t, J = 7.4 Hz, 3H), 7.56 (t, J = 8.0 Hz, 2H), 7.43 (t, J = 7.5 Hz, 2H). ^{13}C NMR (101 MHz, $CDCl_3$) δ 180.04, 143.68, 141.90, 141.45, 140.98, 132.02, 131.36, 131.17, 127.21, 127.13, 124.83, 123.76, 123.45, 121.08, 113.77. HRMS (EI, *m*/*z*). Calcd for $C_{32}H_{21}BN_2O_2$: 472.1383, Found: 472.1375.

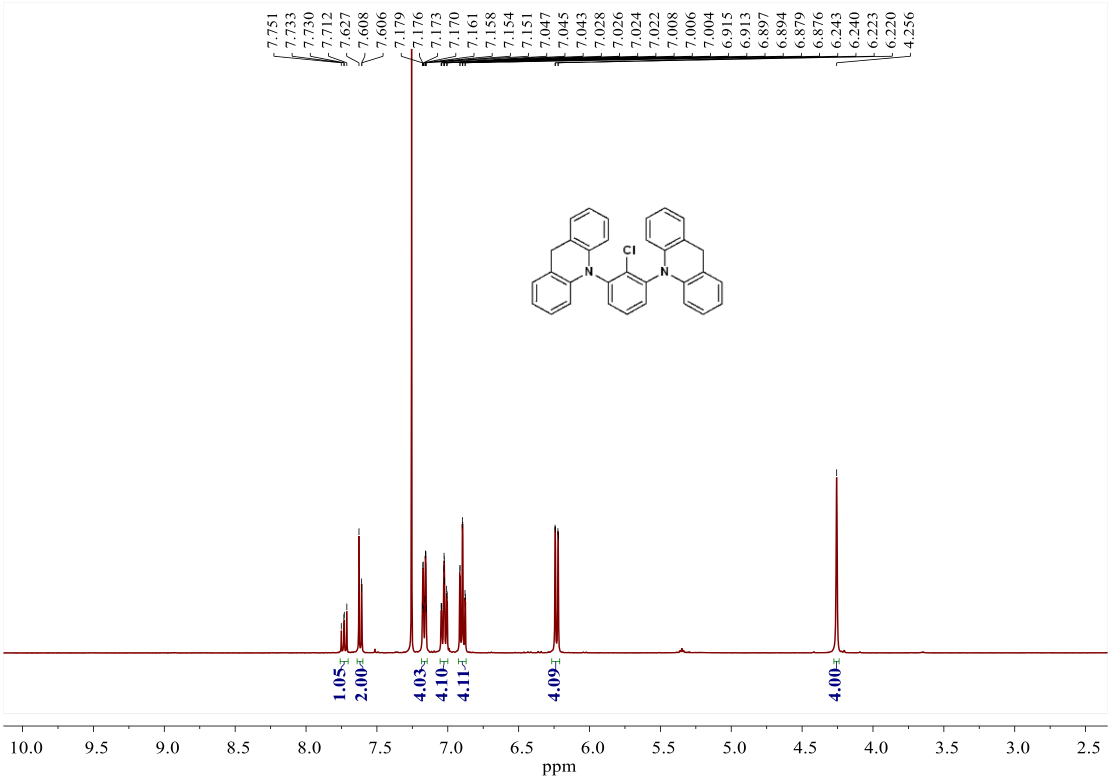

Supplementary Fig. 12. ^{1}H NMR spectrum of 3 in CD_2Cl_3.

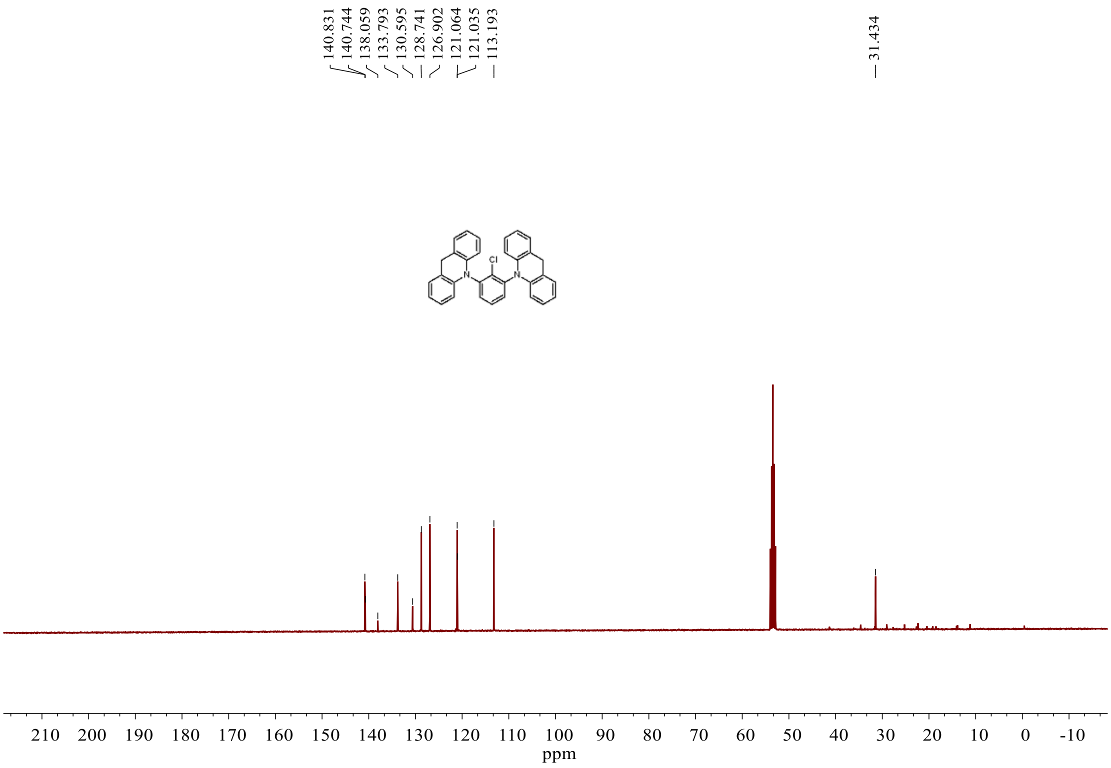

Supplementary Fig. 13. ^{13}C NMR spectrum of 3 in CD_2Cl_2.

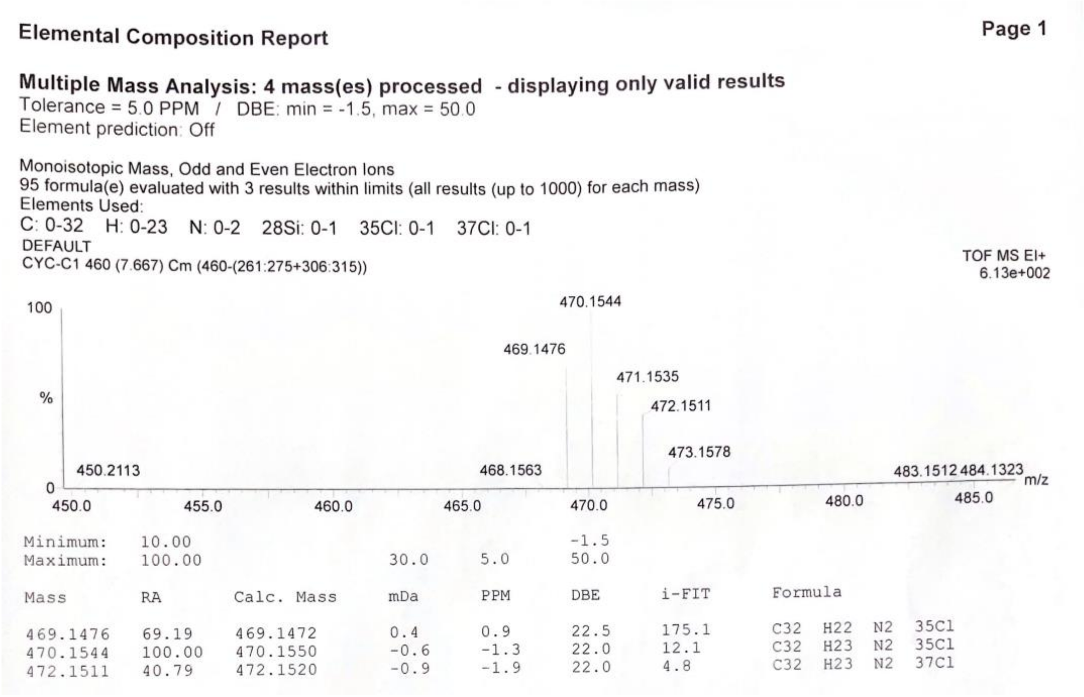

Elemental Composition Report **Page 1**

Multiple Mass Analysis: 4 mass(es) processed - displaying only valid results
Tolerance = 5.0 PPM / DBE: min = -1.5, max = 50.0
Element prediction: Off

Monoisotopic Mass, Odd and Even Electron Ions
95 formula(e) evaluated with 3 results within limits (all results (up to 1000) for each mass)
Elements Used:
C: 0-32 H: 0-23 N: 0-2 28Si: 0-1 35Cl: 0-1 37Cl: 0-1
DEFAULT
CYC-C1 460 (7.667) Cm (460-(261:275+306:315))

TOF MS EI+
6.13e+002

Minimum:	10.00				-1.5		
Maximum:	100.00		30.0	5.0	50.0		
Mass	RA	Calc. Mass	mDa	PPM	DBE	i-FIT	Formula
469.1476	69.19	469.1472	0.4	0.9	22.5	175.1	C32 H22 N2 35Cl
470.1544	100.00	470.1550	-0.6	-1.3	22.0	12.1	C32 H23 N2 35Cl
472.1511	40.79	472.1520	-0.9	-1.9	22.0	4.8	C32 H23 N2 37Cl

Supplementary Fig. 14. The HRMS of compound 3.

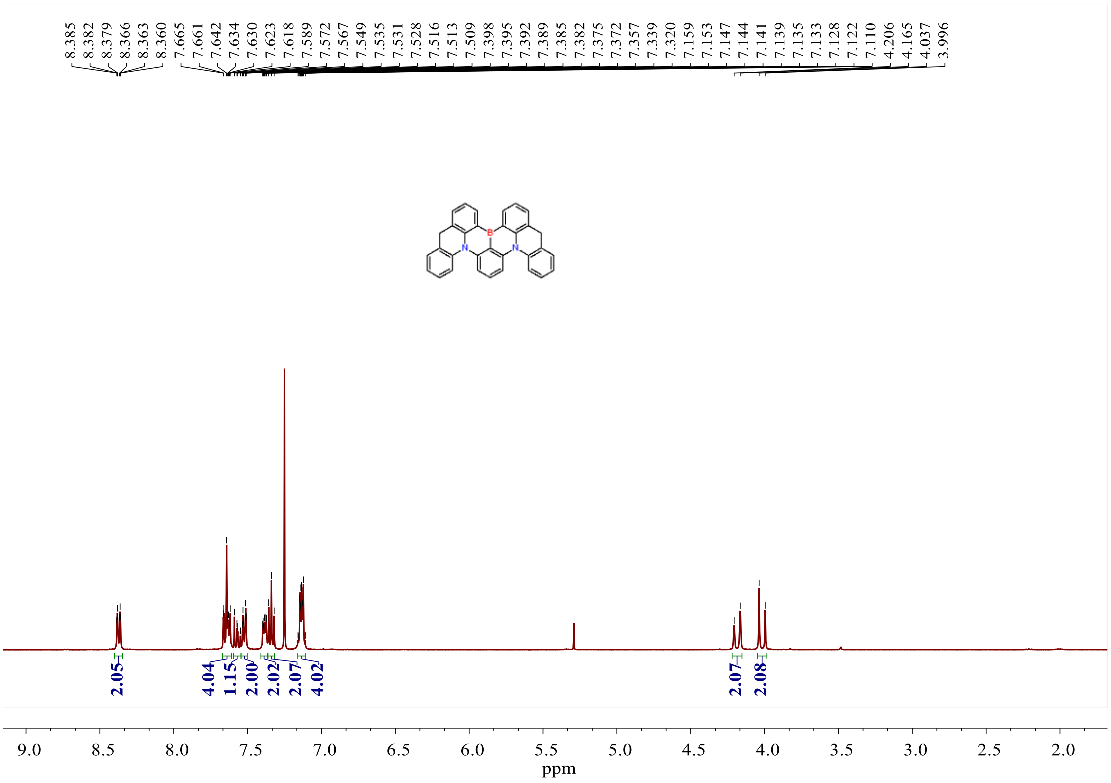

Supplementary Fig. 15. ^{1}H NMR spectrum of 4 in $CDCl_3$.

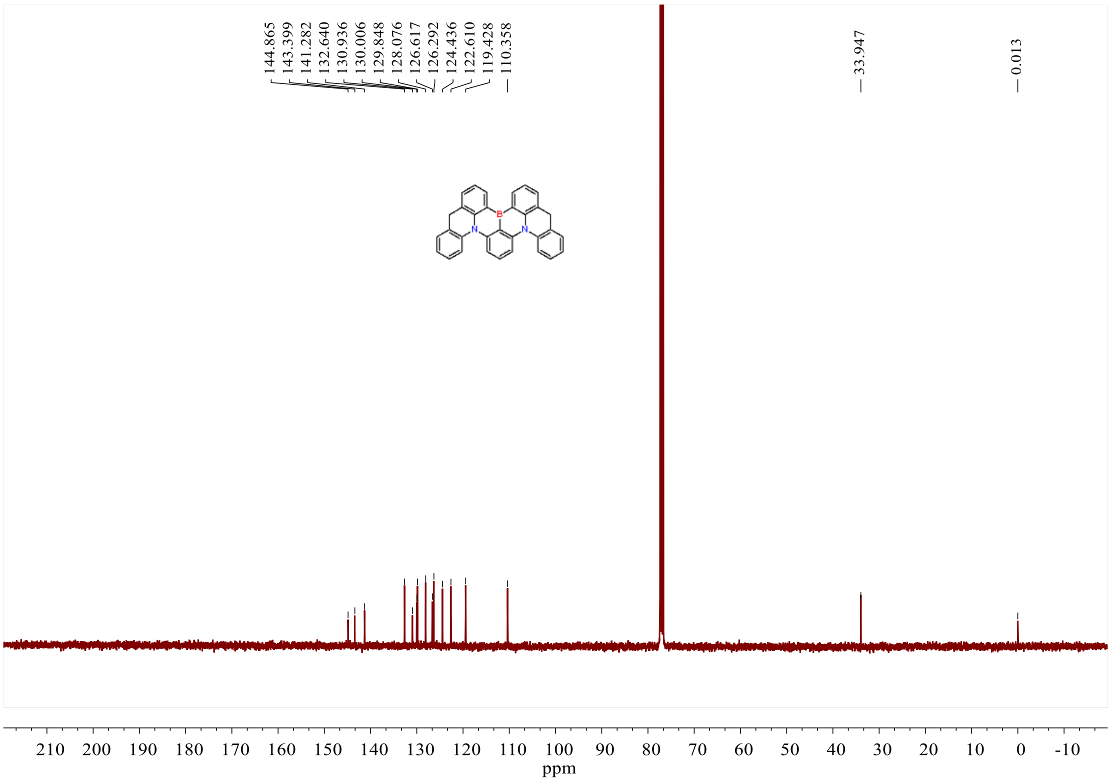

Supplementary Fig. 16. ^{13}C NMR spectrum of 4 in $CDCl_3$.

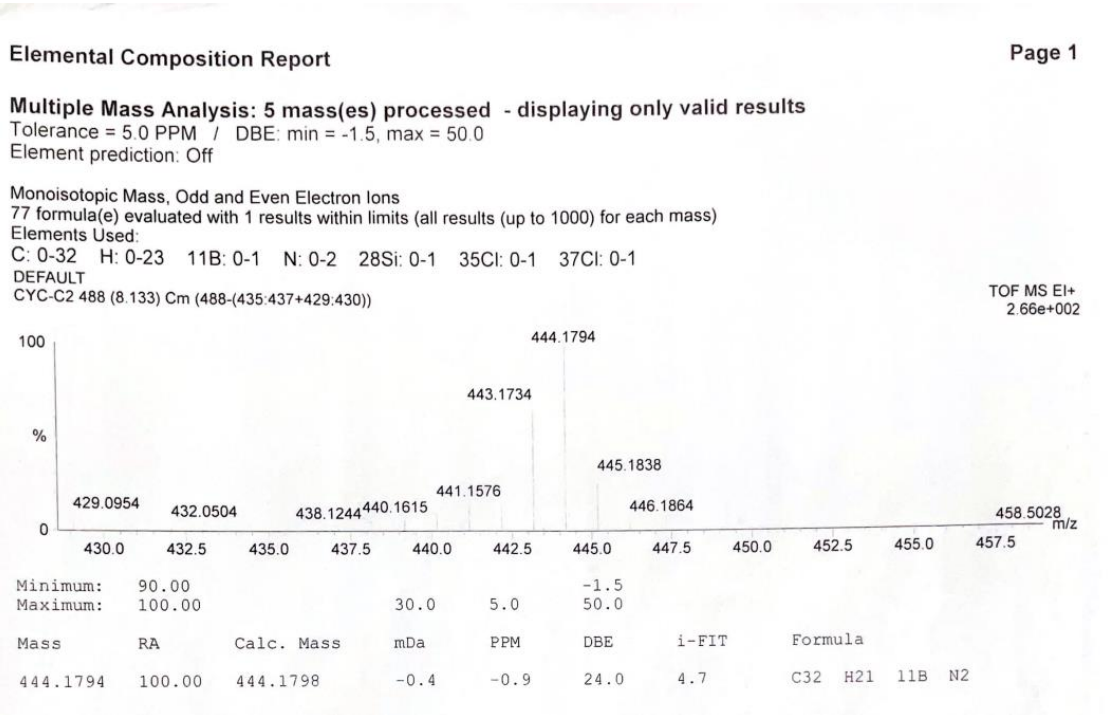

Elemental Composition Report

Page 1

Multiple Mass Analysis: 5 mass(es) processed - displaying only valid results
Tolerance = 5.0 PPM / DBE: min = -1.5, max = 50.0
Element prediction: Off

Monoisotopic Mass, Odd and Even Electron Ions
77 formula(e) evaluated with 1 results within limits (all results (up to 1000) for each mass)
Elements Used:
C: 0-32 H: 0-23 11B: 0-1 N: 0-2 28Si: 0-1 35Cl: 0-1 37Cl: 0-1
DEFAULT
CYC-C2 488 (8.133) Cm (488-(435:437+429:430))

Minimum:	90.00				-1.5		
Maximum:	100.00		30.0	5.0	50.0		
Mass	RA	Calc. Mass	mDa	PPM	DBE	i-FIT	Formula
444.1794	100.00	444.1798	-0.4	-0.9	24.0	4.7	C32 H21 11B N2

Supplementary Fig. 17. The HRMS of compound 4.

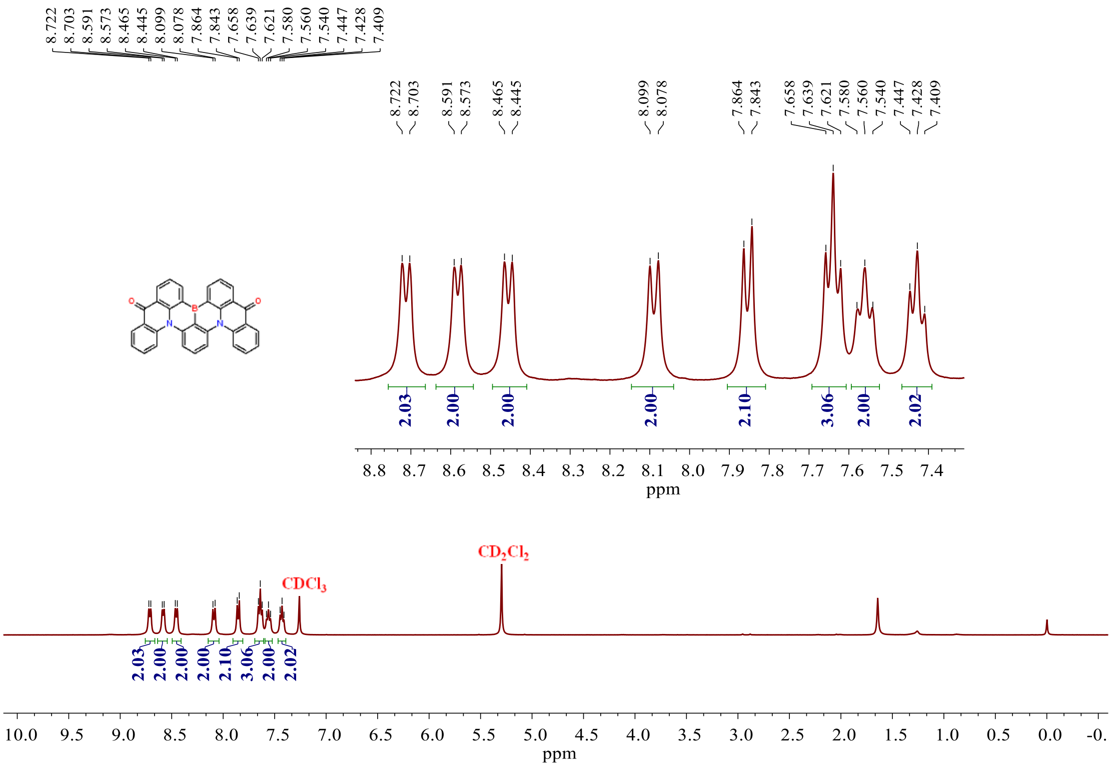

Supplementary Fig. 18. ^{1}H NMR spectrum of *h*-BNCO-1 in CD_2Cl_2:$CDCl_3$ (2:1).

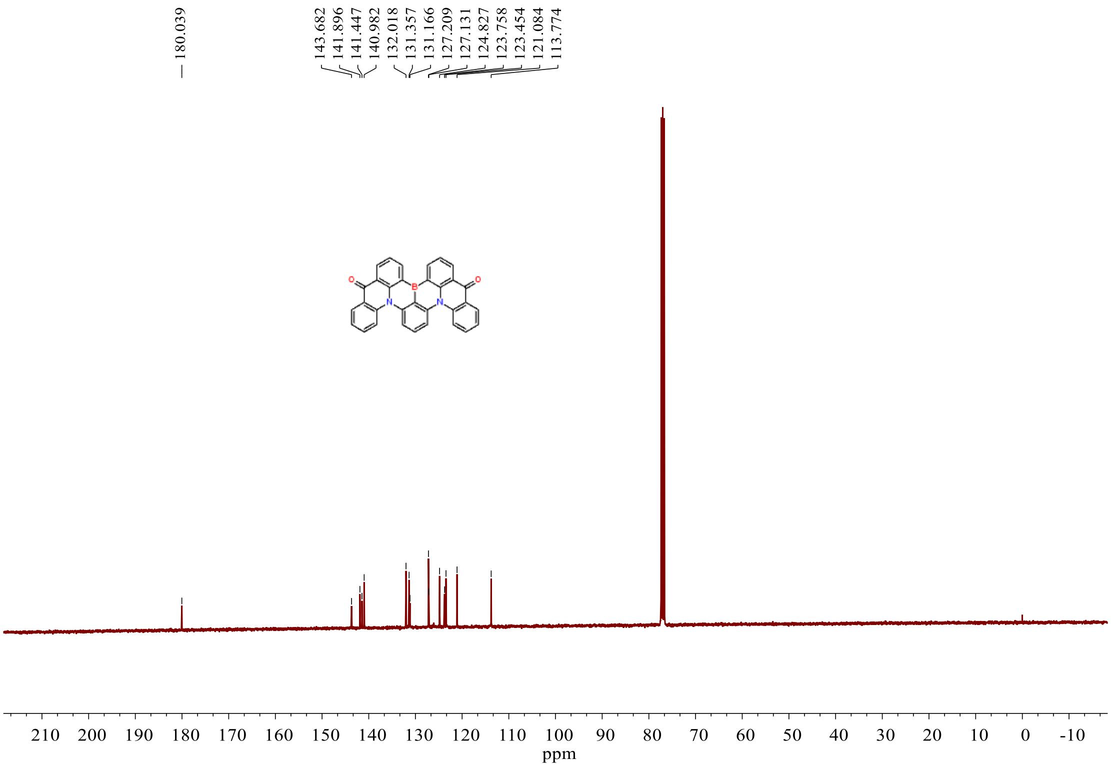

Supplementary Fig. 19. ^{13}C NMR spectrum of *h*-BNCO-1 in $CDCl_3$.

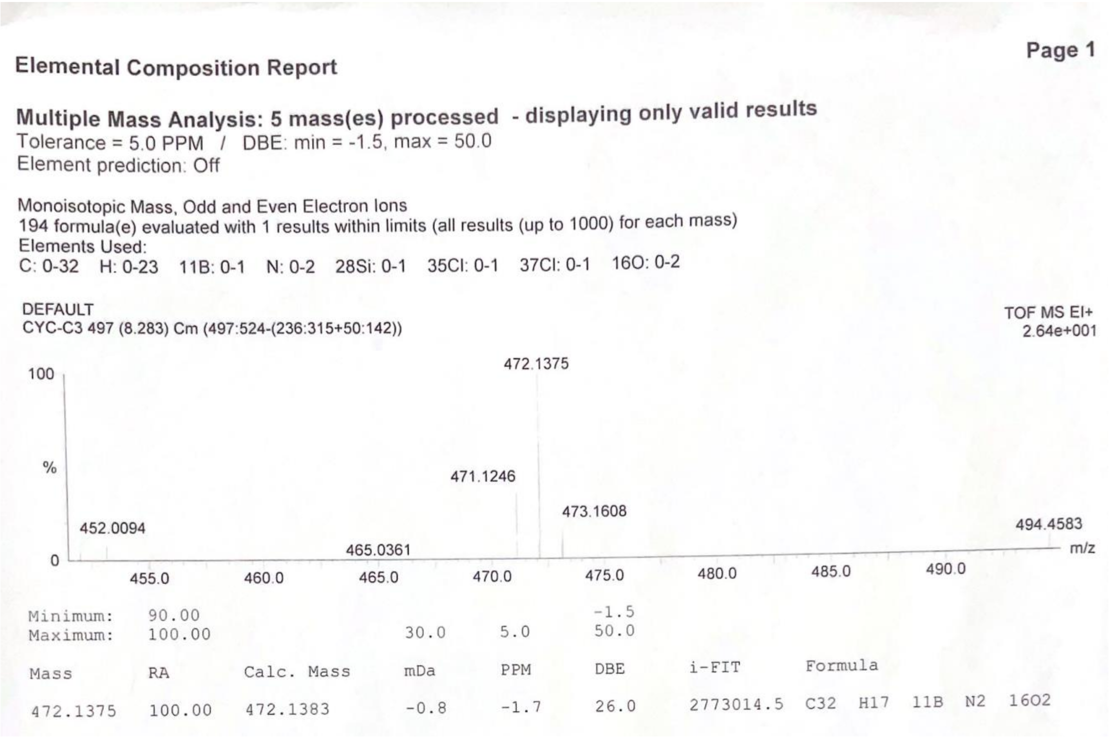

Minimum:	90.00				-1.5		
Maximum:	100.00		30.0	5.0	50.0		
Mass	RA	Calc. Mass	mDa	PPM	DBE	i-FIT	Formula
472.1375	100.00	472.1383	-0.8	-1.7	26.0	2773014.5	C32 H17 11B N2 16O2

Supplementary Fig. 20. The HRMS of *h*-BNCO-1.

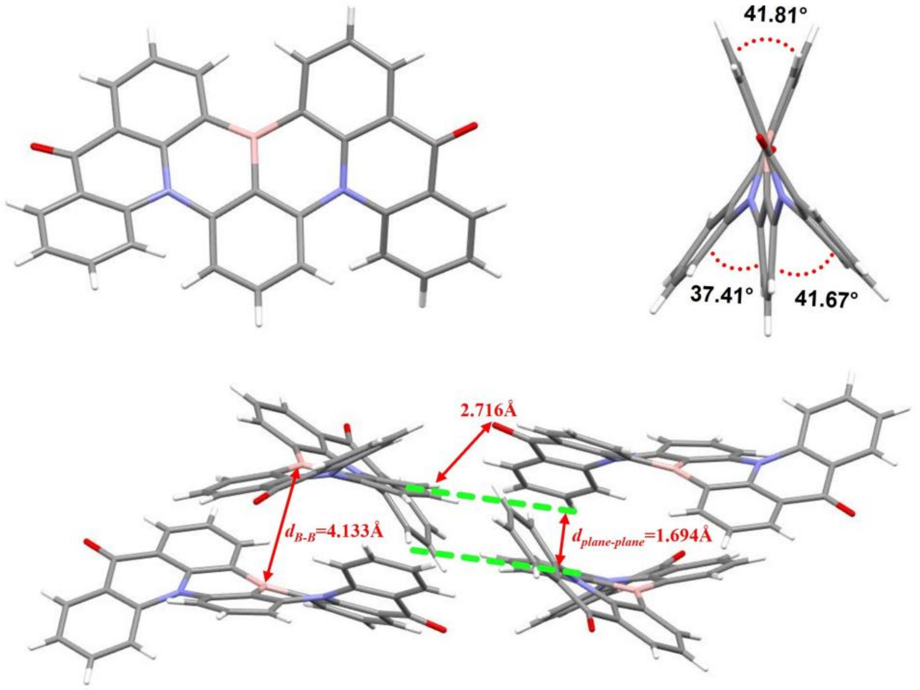

Supplementary Fig. 21. Single crystal structures and packing mode of *h*-BNCO-1 (CCDC: 2224022).

Supplementary Table 4. Crystal data and structure refinement for *h*-BNCO-1.

**Crystal data**	
Empirical formula	$C_{32}H_{17}BN_2O_2$
Formula weight	472.28
Temperature/K	170.0
Crystal system	monoclinic
Space group	$P2_1/c$
a/Å	13.2532(5)
b/Å	20.3310(9)
c/Å	8.0845(3)
α/°	90
β/°	100.5290(10)
γ/°	90
Volume/Å^3	2141.70(15)
Z	4
ρ_{calc}g/cm^3	1.465
μ/mm^{-1}	0.091
F(000)	976.0

Crystal size/mm^3	0.15 × 0.08 × 0.05
Radiation	MoKα (λ = 0.71073)
2Θ range for data collection/°	4.006 to 54.186
Index ranges	$-16 \le h \le 16$, $-22 \le k \le 26$, $-9 \le l \le 10$
Reflections collected	16872
Independent reflections	4684 [R_{int} = 0.0731, R_{sigma} = 0.0746]
Data/restraints/parameters	4684/0/334
Goodness-of-fit on F^2	1.009
Final R indexes [I>=2σ (I)]	R_1 = 0.0557, wR_2 = 0.1383
Final R indexes [all data]	R_1 = 0.0934, wR_2 = 0.1751
Largest diff. peak/hole/e Å^{-3}	0.29/-0.24

Supplementary Table 5. Selected bond lengths and angles (Å, °) from the crystal structure of *h*-BNCO-1.

**Bond lengths**			
O(2)-C(26)	1.231(3)	C(6)-C(7)	1.464(3)
O(1)-C(7)	1.236(3)	C(6)-C(5)	1.400(3)
N(1)-C(12)	1.420(2)	C(2)-C(1)	1.424(3)
N(1)-C(14)	1.431(3)	C(2)-C(3)	1.400(3)
N(1)-C(1)	1.410(3)	C(2)-B(3)	1.526(3)
N(2)-C(20)	1.406(3)	C(10)-C(9)	1.374(3)
N(2)-C(18)	1.431(2)	C(21)-C(22)	1.400(3)
N(2)-C(28)	1.421(3)	C(21)-B(3)	1.529(3)
C(12)-C(8)	1.391(3)	C(28)-C(29)	1.398(3)
C(12)-C(13)	1.400(3)	C(28)-C(27)	1.407(3)
C(8)-C(10)	1.401(3)	C(3)-C(4)	1.385(3)
C(8)-C(7)	1.465(3)	C(5)-C(4)	1.379(3)
C(19)-C(14)	1.412(3)	C(25)-C(26)	1.464(4)
C(19)-C(18)	1.409(3)	C(25)-C(24)	1.394(3)
C(19)-B(3)	1.510(3)	C(26)-C(27)	1.463(4)
C(15)-C(14)	1.396(3)	C(29)-C(30)	1.375(3)
C(15)-C(16)	1.384(3)	C(11)-C(9)	1.382(3)
C(20)-C(21)	1.418(3)	C(27)-C(32)	1.399(3)
C(20)-C(25)	1.412(3)	C(30)-C(31)	1.396(4)
C(13)-C(11)	1.379(3)	C(24)-C(23)	1.378(4)
C(18)-C(17)	1.389(3)	C(22)-C(23)	1.387(3)
C(17)-C(16)	1.384(3)	C(32)-C(31)	1.364(4)
C(6)-C(1)	1.406(3)		
**Bond angles**			
C(12)-N(1)-C(14)	121.25(16)	C(22)-C(21)-B(3)	123.8(2)
C(1)-N(1)-C(12)	118.10(17)	C(15)-C(16)-C(17)	122.4(2)
C(1)-N(1)-C(14)	120.63(16)	C(29)-C(28)-N(2)	121.2(2)
C(20)-N(2)-C(18)	120.81(17)	C(29)-C(28)-C(27)	118.8(2)
C(20)-N(2)-C(28)	118.47(17)	C(27)-C(28)-N(2)	119.8(2)
C(28)-N(2)-C(18)	120.70(18)	N(1)-C(1)-C(2)	120.31(19)

C(8)-C(12)-N(1)	120.31(18)	C(6)-C(1)-N(1)	119.74(18)
C(8)-C(12)-C(13)	118.38(18)	C(6)-C(1)-C(2)	119.94(19)
C(13)-C(12)-N(1)	121.29(19)	O(1)-C(7)-C(8)	122.0(2)
C(12)-C(8)-C(10)	120.4(2)	O(1)-C(7)-C(6)	122.9(2)
C(12)-C(8)-C(7)	120.75(18)	C(6)-C(7)-C(8)	115.11(19)
C(10)-C(8)-C(7)	118.9(2)	C(4)-C(3)-C(2)	122.7(2)
C(14)-C(19)-B(3)	120.24(19)	C(4)-C(5)-C(6)	121.0(2)
C(18)-C(19)-C(14)	118.99(19)	C(20)-C(25)-C(26)	122.1(2)
C(18)-C(19)-B(3)	120.77(18)	C(24)-C(25)-C(20)	118.7(2)
C(16)-C(15)-C(14)	118.72(19)	C(24)-C(25)-C(26)	119.2(2)
N(2)-C(20)-C(21)	120.52(18)	O(2)-C(26)-C(25)	122.0(2)
N(2)-C(20)-C(25)	119.1(2)	O(2)-C(26)-C(27)	123.1(2)
C(25)-C(20)-C(21)	120.4(2)	C(27)-C(26)-C(25)	114.71(19)
C(19)-C(14)-N(1)	118.43(18)	C(30)-C(29)-C(28)	120.5(2)
C(15)-C(14)-N(1)	121.61(17)	C(5)-C(4)-C(3)	119.0(2)
C(15)-C(14)-C(19)	119.94(19)	C(13)-C(11)-C(9)	121.4(2)
C(11)-C(13)-C(12)	120.2(2)	C(28)-C(27)-C(26)	121.0(2)
C(19)-C(18)-N(2)	118.38(18)	C(32)-C(27)-C(28)	119.2(2)
C(17)-C(18)-N(2)	121.16(19)	C(32)-C(27)-C(26)	119.3(2)
C(17)-C(18)-C(19)	120.42(18)	C(10)-C(9)-C(11)	118.9(2)
C(16)-C(17)-C(18)	118.7(2)	C(29)-C(30)-C(31)	120.5(3)
C(1)-C(6)-C(7)	120.80(19)	C(23)-C(24)-C(25)	121.9(2)
C(5)-C(6)-C(1)	119.7(2)	C(23)-C(22)-C(21)	122.4(2)
C(5)-C(6)-C(7)	119.5(2)	C(24)-C(23)-C(22)	118.8(2)
C(1)-C(2)-B(3)	118.00(19)	C(31)-C(32)-C(27)	121.3(2)
C(3)-C(2)-C(1)	117.4(2)	C(32)-C(31)-C(30)	119.4(2)
C(3)-C(2)-B(3)	124.43(19)	C(19)-B(3)-C(2)	115.01(19)

C(9)-C(10)-C(8)	120.6(2)	C(19)-B(3)-C(21)	114.5(2)
C(20)-C(21)-B(3)	118.4(2)	C(2)-B(3)-C(21)	130.5(2)
C(22)-C(21)-C(20)	117.6(2)		

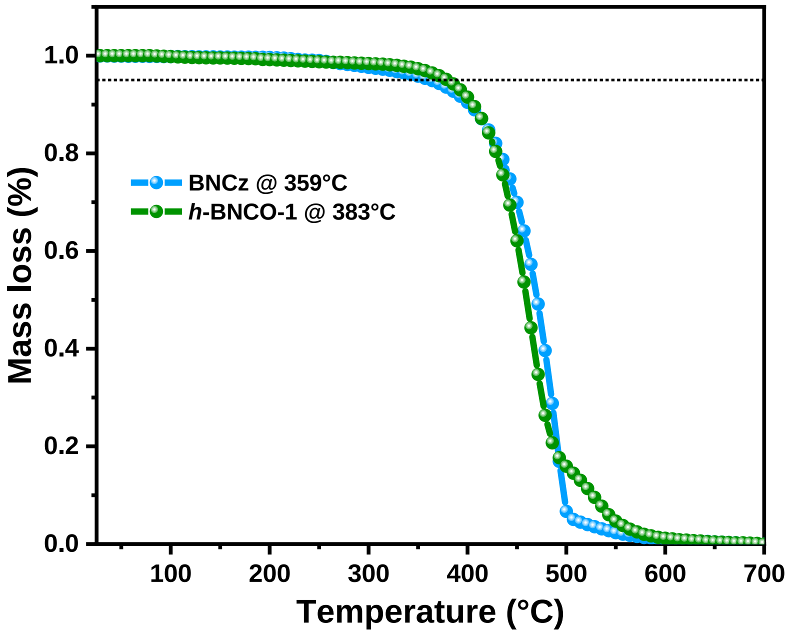

Supplementary Fig. 22. Thermogravimetric analysis curves of BNCZ and *h*-BNCO-1.

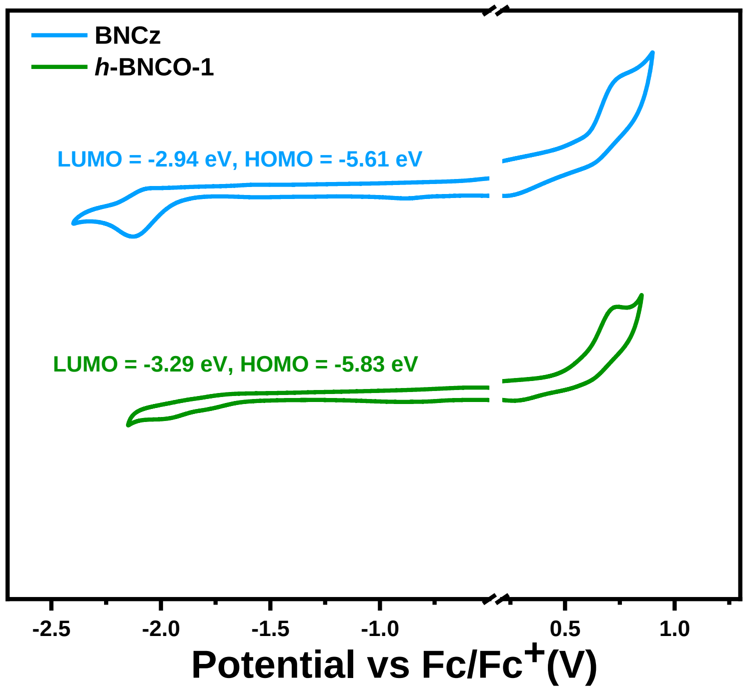

Supplementary Fig. 23. Cyclic voltammetry curves of BNCZ and *h*-BNCO-1 in DMF.

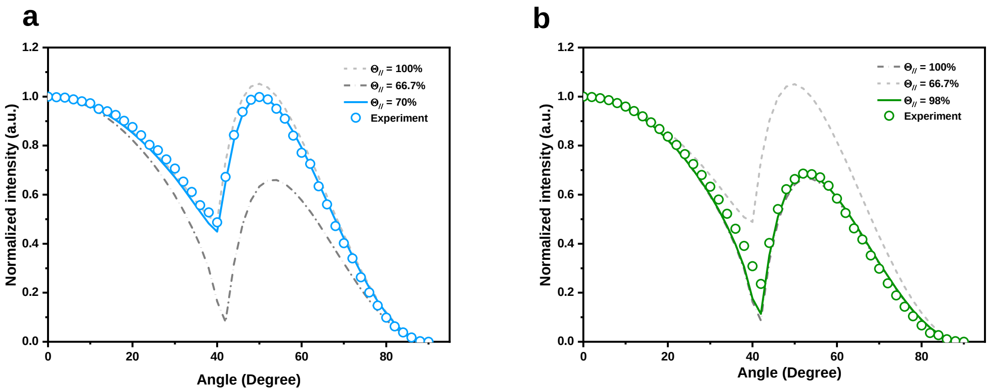

Supplementary Fig. 24. Characterization of the horizontal dipole orientation by angle-dependent PL intensity of the p-polarized light. Measured horizontal transition dipole moment ratios for **a**, BNCZ and **b**, *h*-BNCO-1 1 wt%-doped in DMIC-TRZ films.

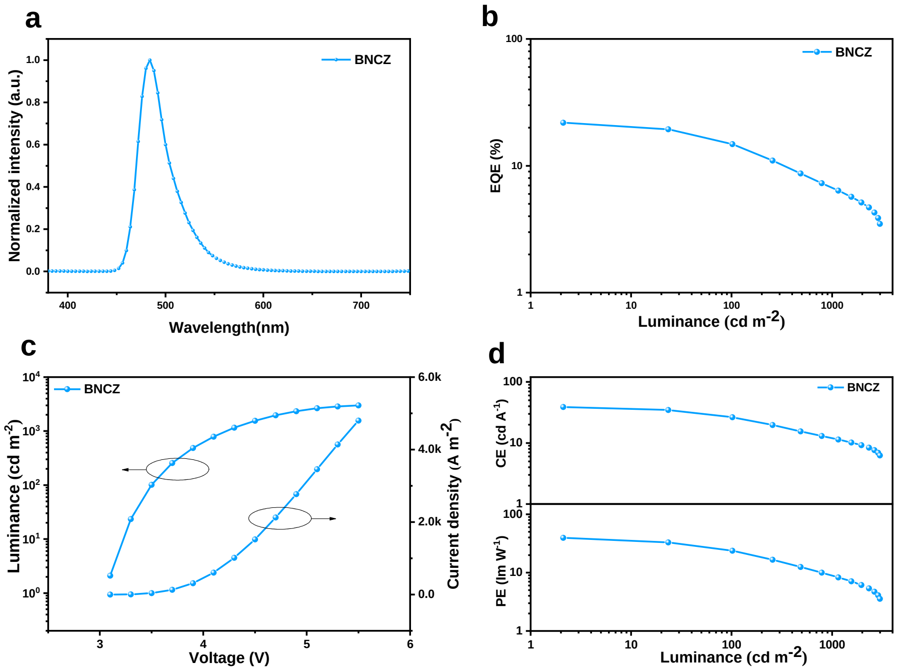

Supplementary Fig. 25. **a**, Normalized EL spectra. **b**, EQE−luminance characteristics. **c**, Luminance−voltage−current density characteristics. **d**, CE−luminance and PE−luminance characteristics of the OLED with a structure of ITO /HAT-CN (7 nm)/TAPC (30 nm)/TCTA (10 nm)/mCBP (10 nm)/PhCzBCz: 1 wt% BNCZ (20 nm)/TmPyPB (40 nm)/LiF (1 nm)/Al. The device exhibits a EL maximum at 484 nm with an FWHM of 34 nm/178 meV, corresponding to CIE coordinates of (0.11, 0.34), operates a maximum EQE of 21.8%, a maximum CE of 39.0 cd A^{-1} and maximum PE of 39.5 lm W^{-1}, respectively, and maintains an EQE of 6.63 % at 1000 cd m^{-2}.

References

1 Li, X. *et al.* Thermally Activated Delayed Fluorescence Carbonyl Derivatives for Organic Light-Emitting Diodes with Extremely Narrow Full Width at Half-Maximum. *ACS Appl. Mater. Interfaces* **11**, 13472-13480 (2019).

2 Huang, F. *et al.* Approaching Efficient and Narrow RGB Electroluminescence from D-A-Type TADF Emitters Containing an Identical Multiple Resonance Backbone as the Acceptor. *ACS Appl. Mater. Interfaces* **13**, 36089-36097 (2021).

3 Fan, X. C. *et al.* Managing Intersegmental Charge-Transfer and Multiple Resonance Alignments of D_3-A Typed TADF Emitters for Red OLEDs with Improved Efficiency and Color Purity. *Adv. Opt. Mater.* **10**, 2101789 (2021).

4 Hall, D. *et al.* Improving Processability and Efficiency of Resonant TADF Emitters: A Design Strategy. *Adv. Opt. Mater.* **8**, 1901627 (2019).

5 Wu, S. *et al.* Excited-State Modulation in Donor-Substituted Multiresonant Thermally Activated Delayed Fluorescence Emitters. *ACS Appl. Mater. Interfaces* **14**, 22341-22352 (2022).

6 Wu, S. *et al.* Highly Efficient Green and Red Narrowband Emissive Organic Light-Emitting Diodes Employing Multi-Resonant Thermally Activated Delayed Fluorescence Emitters. *Angew. Chem. Int. Ed.* **61**, e202213697 (2022).

7 Yuan, Y. *et al.* The Design of Fused Amine/Carbonyl System for Efficient Thermally Activated Delayed Fluorescence: Novel Multiple Resonance Core and Electron Acceptor. *Adv. Opt. Mater.* **7**, 1801536 (2019).

8 Zou, S. N. *et al.* Fully Bridged Triphenylamine Derivatives as Color-Tunable Thermally Activated Delayed Fluorescence Emitters. *Org. Lett.* **23**, 958-962 (2021).

9 Qiu, X. *et al.* Narrowband Emission from Organic Fluorescent Emitters with Dominant Low-Frequency Vibronic Coupling. *Adv. Opt. Mater.* **9**, 2001845 (2020).

10 Min, H., Park, I. S. & Yasuda, T. cis-Quinacridone-Based Delayed Fluorescence Emitters: Seemingly Old but Renewed Functional Luminogens. *Angew. Chem. Int. Ed.* **60**, 7643-7648 (2021).

11 Hatakeyama, T. *et al.* Ultrapure Blue Thermally Activated Delayed Fluorescence Molecules: Efficient HOMO-LUMO Separation by the Multiple Resonance Effect. *Adv. Mater.* **28**, 2777-2781 (2016).

12 Wang, Y. *et al.* A periphery cladding strategy to improve the performance of narrowband emitters, achieving deep-blue OLEDs with CIEy < 0.08 and external quantum efficiency approaching 20%. *Org. Electron.* **97**, 106275 (2021).

13 Wang, Y. *et al.* The selective regulation of borylation site based on one-shot electrophilic C–H borylation reaction, achieving highly efficient narrowband organic light-emitting diodes. *Chem. Eng. J.* **431**, 133221 (2022).

14 Matsui, K. *et al.* One-Shot Multiple Borylation toward BN-Doped Nanographenes. *J. Am. Chem. Soc.* **140**, 1195-1198 (2018).

15 Kondo, Y. *et al.* Narrowband deep-blue organic light-emitting diode featuring an organoboron-based emitter. *Nat. Photonics.* **13**, 678-682 (2019).

16 Ikeda, N. *et al.* Solution-Processable Pure Green Thermally Activated Delayed Fluorescence Emitter Based on the Multiple Resonance Effect. *Adv. Mater.* **32**, e2004072 (2020).

17 Oda, S. *et al.* Carbazole-Based DABNA Analogues as Highly Efficient Thermally Activated

Delayed Fluorescence Materials for Narrowband Organic Light-Emitting Diodes. *Angew. Chem. Int. Ed.* **60**, 2882-2886 (2021).
18 Tanaka, H. *et al.* Hypsochromic Shift of Multiple-Resonance-Induced Thermally Activated Delayed Fluorescence by Oxygen Atom Incorporation. *Angew. Chem. Int. Ed.* **60**, 17910-17914 (2021).
19 Oda, S. *et al.* One-Shot Synthesis of Expanded Heterohelicene Exhibiting Narrowband Thermally Activated Delayed Fluorescence. *J. Am. Chem. Soc.* **144**, 106-112 (2022).
20 Oda, S. *et al.* Ultra-Narrowband Blue Multi-Resonance Thermally Activated Delayed Fluorescence Materials. *Adv. Sci.* **10**, e2205070 (2022).
21 Oda, S. *et al.* Development of Pure Green Thermally Activated Delayed Fluorescence Material by Cyano Substitution. *Adv. Mater.* **34**, e2201778 (2022).
22 Uemura, S. *et al.* Sequential Multiple Borylation Toward an Ultrapure Green Thermally Activated Delayed Fluorescence Material. *J. Am. Chem. Soc.* **145**, 1505-1511 (2022).
23 Huang, F. *et al.* Distinguishing the respective determining factors for spectral broadening and concentration quenching in multiple resonance type TADF emitter systems. *Mater Horiz* **9**, 2226-2232 (2022).
24 Cheng, Y. C. *et al.* A Highly Twisted Carbazole-Fused DABNA Derivative as an Orange-Red TADF Emitter for OLEDs with Nearly 40 % EQE. *Angew. Chem. Int. Ed.* **61**, e202212575 (2022).
25 Fan, X.-C. *et al.* Ultrapure green organic light-emitting diodes based on highly distorted fused π-conjugated molecular design. *Nat. Photonics* (2023) DOI: 10.1038/s41566-022-01106-8.
26 Liu, F. *et al.* Highly Efficient Multi-Resonance Thermally Activated Delayed Fluorescence Material with a Narrow Full Width at Half-Maximum of 0.14 eV. *Small* **18**, e2106462 (2022).
27 Liu, F. *et al.* Highly Efficient Asymmetric Multiple Resonance Thermally Activated Delayed Fluorescence Emitter with EQE of 32.8 % and Extremely Low Efficiency Roll-Off. *Angew. Chem. Int. Ed.* **61**, e202116927 (2022).
28 Zhang, Y. *et al.* Multi-Resonance Induced Thermally Activated Delayed Fluorophores for Narrowband Green OLEDs. *Angew. Chem. Int. Ed.* **58**, 16912-16917 (2019).
29 Zhang, Y. *et al.* Achieving Pure Green Electroluminescence with CIEy of 0.69 and EQE of 28.2% from an Aza-Fused Multi-Resonance Emitter. *Angew. Chem. Int. Ed.* **59**, 17499-17503 (2020).
30 Zhang, Y. *et al.* Fusion of Multi-Resonance Fragment with Conventional Polycyclic Aromatic Hydrocarbon for Nearly BT.2020 Green Emission. *Angew. Chem. Int. Ed.* **61**, e202202380 (2022).
31 Wang, X. *et al.* Mesityl-Functionalized Multi-Resonance Organoboron Delayed Fluorescent Frameworks with Wide-Range Color Tunability for Narrowband OLEDs. *Angew. Chem. Int. Ed.* **61**, e202206916 (2022).
32 Xu, Y. *et al.* Constructing Charge-Transfer Excited States Based on Frontier Molecular Orbital Engineering: Narrowband Green Electroluminescence with High Color Purity and Efficiency. *Angew. Chem. Int. Ed.* **59**, 17442-17446 (2020).
33 Zhang, Y. *et al.* Sterically Wrapped Multiple Resonance Fluorophors for Suppression of Concentration Quenching and Spectrum Broadening. *Angew. Chem. Int. Ed.* **61**, e202113206 (2022).
34 Zhang, Y. *et al.* Multi-Resonance Deep-Red Emitters with Shallow Potential-Energy Surfaces to Surpass Energy-Gap Law*. *Angew. Chem. Int. Ed.* **60**, 20498-20503 (2021).

35 Fan, T. *et al.* One-Shot Synthesis of B/N-Doped Calix[4]arene Exhibiting Narrowband Multiple Resonance Fluorescence. *Angew. Chem. Int. Ed.* **61**, e202213585 (2022).
36 Meng, G. *et al.* Amine-Directed Formation of B-N Bonds for BN-Fused Polycyclic Aromatic Multiple Resonance Emitters with Narrowband Emission. *Angew. Chem. Int. Ed.* **61**, e202207293 (2022).
37 Xu, Y. *et al.* Molecular-Structure and Device-Configuration Optimizations toward Highly Efficient Green Electroluminescence with Narrowband Emission and High Color Purity. *Adv. Opt. Mater.* **8**, 1902142 (2020).
38 Xu, Y., Wang, Q., Cai, X., Li, C. & Wang, Y. Highly Efficient Electroluminescence from Narrowband Green Circularly Polarized Multiple Resonance Thermally Activated Delayed Fluorescence Enantiomers. *Adv. Mater.* **33**, e2100652 (2021).
39 Xu, Y. *et al.* Highly Efficient Electroluminescent Materials with High Color Purity Based on Strong Acceptor Attachment onto B–N-Containing Multiple Resonance Frameworks. *CCS Chem.* **4**, 2065-2079 (2022).
40 Xu, Y. *et al.* Constructing Organic Electroluminescent Material with Very High Color Purity and Efficiency Based on Polycyclization of the Multiple Resonance Parent Core. *Angew. Chem. Int. Ed.* **61**, e202204652 (2022).
41 Cai, X. *et al.* Achieving 37.1% Green Electroluminescent Efficiency and 0.09 eV Full Width at Half Maximum Based on a 3 Boron-Oxygen-Nitrogen Embedded Polycyclic Aromatic System. *Angew. Chem. Int. Ed.* **61**, e202200337 (2022).
42 Wang, Q., Xu, Y., Yang, T., Xue, J. & Wang, Y. Precise Functionalization of Multiple Resonance Framework: Constructing Narrowband Organic Electroluminescent Materials with External Quantum Efficiency over 40. *Adv. Mater.*, **35**, e2205166 (2023).
43 Cai, X. *et al.* Solution-Processable Pure-Red Multiple Resonance-induced Thermally Activated Delayed Fluorescence Emitter for Organic Light-Emitting Diode with External Quantum Efficiency over 20%. *Angew. Chem. Int. Ed.* **62**, e202216473 (2023).
44 Yan, X. *et al.* Achieving highly efficient narrowband sky-blue electroluminescence with alleviated efficiency roll-off by molecular-structure regulation and device-configuration optimization. *J. Mater. Chem. C* **10**, 15408-15415 (2022).
45 Kim, J. H., Chung, W. J., Kim, J. & Lee, J. Y. Concentration quenching–resistant multiresonance thermally activated delayed fluorescence emitters. *Mater. Today Energy* **21**, 100792 (2021).
46 Park, J., Kim, K. J., Lim, J., Kim, T. & Lee, J. Y. High Efficiency of over 25% and Long Device Lifetime of over 500 h at 1000 nit in Blue Fluorescent Organic Light-Emitting Diodes. *Adv. Mater.* **34**, e2108581 (2022).
47 Park, J. *et al.* Fine-tuned asymmetric blue multiple resonance thermally activated delayed fluorescence emitters with high efficiency and narrow emission band. *J. Mater. Chem. C.* **10**, 12300-12306 (2022).
48 Qi, Y. *et al.* Peripheral Decoration of Multi-Resonance Molecules as a Versatile Approach for Simultaneous Long-Wavelength and Narrowband Emission. *Adv. Funct. Mater.* **31**, 2102017 (2021).
49 Qiu, Y. *et al.* Narrowing the Electroluminescence Spectra of Multiresonance Emitters for High-Performance Blue OLEDs by a Peripheral Decoration Strategy. *ACS Appl. Mater. Interfaces* **13**, 59035-59042 (2021).
50 Jiang, P. *et al.* Simple Acridan-Based Multi-Resonance Structures Enable Highly Efficient

Narrowband Green TADF Electroluminescence. *Adv. Opt. Mater.* **9**, 2100825 (2021).
51 Chen, Z. *et al.* High-Performance Circularly Polarized Electroluminescence with Simultaneous Narrowband Emission, High Efficiency, and Large Dissymmetry Factor. *Adv. Mater.* **34**, e2109147 (2022).
52 Jiang, P. *et al.* Quenching-Resistant Multiresonance TADF Emitter Realizes 40% External Quantum Efficiency in Narrowband Electroluminescence at High Doping Level. *Adv. Mater.* **34**, e2106954 (2022).
53 Yang, Y. *et al.* Chiral Multi-Resonance TADF Emitters Exhibiting Narrowband Circularly Polarized Electroluminescence with an EQE of 37.2%. *Angew. Chem. Int. Ed.* **61**, e202202227 (2022).
54 Lv, X. *et al.* Extending the pi-Skeleton of Multi-Resonance TADF Materials towards High-Efficiency Narrowband Deep-Blue Emission. *Angew. Chem. Int. Ed.* **61**, e202201588 (2022).
55 Liu, G. *et al.* Facile synthesis of multi-resonance ultra-pure-green TADF emitters based on bridged diarylamine derivatives for efficient OLEDs with narrow emission. *J. Mater. Chem. C* **9**, 8308-8313 (2021).
56 Meng, G. *et al.* Multi-resonant thermally activated delayed fluorescence emitters based on tetracoordinate boron-containing PAHs: colour tuning based on the nature of chelates. *Chem. Sci.* **13**, 1665-1674 (2022).
57 Lee, H. *et al.* Manipulating Spectral Width and Emission Wavelength towards Highly Efficient Blue Asymmetric Carbazole Fused Multi-Resonance Emitters. *ACS Appl. Mater. Interfaces* **14**, 36927-36935 (2022).
58 Rayappa Naveen, K. *et al.* Deep blue diboron embedded multi-resonance thermally activated delayed fluorescence emitters for narrowband organic light emitting diodes. *Chem. Eng. J.* **432**, 134381 (2022).
59 Yang, M., Park, I. S. & Yasuda, T. Full-Color, Narrowband, and High-Efficiency Electroluminescence from Boron and Carbazole Embedded Polycyclic Heteroaromatics. *J. Am. Chem. Soc.* **142**, 19468-19472 (2020).
60 Yang, M. *et al.* Wide-Range Color Tuning of Narrowband Emission in Multi-resonance Organoboron Delayed Fluorescence Materials through Rational Imine/Amine Functionalization. *Angew. Chem. Int. Ed.* **60**, 23142-23147 (2021).
61 Cheon, H. J., Woo, S. J., Baek, S. H., Lee, J. H. & Kim, Y. H. Dense Local Triplet States and Steric Shielding of a Multi-Resonance TADF Emitter Enable High-Performance Deep-Blue OLEDs. *Adv. Mater.* **34**, e2207416 (2022).
62 Cheon, H. J., Shin, Y. S., Park, N. H., Lee, J. H. & Kim, Y. H. Boron-Based Multi-Resonance TADF Emitter with Suppressed Intermolecular Interaction and Isomer Formation for Efficient Pure Blue OLEDs. *Small* **18**, e2107574 (2022).
63 Wu, X. *et al.* Fabrication of Circularly Polarized MR-TADF Emitters with Asymmetrical Peripheral-Lock Enhancing Helical B/N-Doped Nanographenes. *Adv. Mater.* **34**, e2105080 (2022).
64 Hu, J.-J. *et al.* Green multi-resonance thermally activated delayed fluorescence emitters containing phenoxazine units with highly efficient electroluminescence. *J. Mater. Chem. C.* **10**, 768-773 (2022).
65 Luo, X. F. *et al.* High-Efficiency and Narrowband OLEDs from Blue to Yellow with 3 Boron/Nitrogen-Based Polycyclic Heteroaromatic Emitters. *Adv. Opt. Mater.* **10**, 2200504

(2022).

66 Qu, Y. K. *et al.* Steric Modulation of Spiro Structure for Highly Efficient Multiple Resonance Emitters. *Angew. Chem. Int. Ed.* **61**, e202201886 (2022).

67 Liu, Y., Xiao, X., Ran, Y., Bin, Z. & You, J. Molecular design of thermally activated delayed fluorescent emitters for narrowband orange-red OLEDs boosted by a cyano-functionalization strategy. *Chem. Sci.* **12**, 9408-9412 (2021).

68 Liu, Y. *et al.* Space-Confined Donor-Acceptor Strategy Enables Fast Spin-Flip of Multiple Resonance Emitters for Suppressing Efficiency Roll-Off. *Angew. Chem. Int. Ed.* **61**, e202210210 (2022).

69 Luo, X. F. *et al.* Fused π-Extended Multiple-Resonance Induced Thermally Activated Delayed Fluorescence Materials for High-Efficiency and Narrowband OLEDs with Low Efficiency Roll-Off. *Adv. Opt. Mater.* **10**, 2102513 (2022).

70 Liu, J. *et al.* Toward a BT.2020 green emitter through a combined multiple resonance effect and multi-lock strategy. *Nat. Commun.* **13**, 4876 (2022).

71 Madayanad Suresh, S. *et al.* A Deep-Blue-Emitting Heteroatom-Doped MR-TADF Nonacene for High-Performance Organic Light-Emitting Diodes. *Angew. Chem. Int. Ed.* **62**, e202215522 (2022).

72 Luo, X. F. *et al.* Multiple-Resonance-Induced Thermally Activated Delayed Fluorescence Materials Based on Indolo[3,2,1-jk]carbazole with an Efficient Narrowband Pure-Green Electroluminescence. *Angew. Chem. Int. Ed.* **61**, e202209984 (2022).

73 Park, J. *et al.* Asymmetric Blue Multiresonance TADF Emitters with a Narrow Emission Band. *ACS Appl. Mater. Interfaces* **13**, 45798-45805 (2021).

74 Xiong, X., Cheng, Y.-C., Wang, K., Yu, J. & Zhang, X. A comparative study of two multi-resonance TADF analogous materials integrating chalcogen atoms of different periods. *Mater. Chem. Front.* (2023) DOI: 10.1039/d2qm01304e.

75 Cai, S. *et al.* Gold(I) Multi-Resonance Thermally Activated Delayed Fluorescent Emitters for Highly Efficient Ultrapure-Green Organic Light-Emitting Diodes. *Angew. Chem. Int. Ed.*, **61**, e202213392 (2022).

76 Liang, X. *et al.* Peripheral Amplification of Multi-Resonance Induced Thermally Activated Delayed Fluorescence for Highly Efficient OLEDs. *Angew. Chem. Int. Ed.* **57**, 11316-11320 (2018).

77 Han, J. *et al.* Simple Molecular Design Strategy for Multiresonance Induced TADF Emitter: Highly Efficient Deep Blue to Blue Electroluminescence with High Color Purity. *Adv. Opt. Mater.* **10**, 2102092 (2021).

78 Hua, T. *et al.* Sulfone-Incorporated Multi-Resonance TADF Emitter for High-Performance Narrowband Blue OLEDs with EQE of 32%. *Adv. Funct. Mater.* **32**, 2201032 (2022).

79 Nagata, M. *et al.* Fused-Nonacyclic Multi-Resonance Delayed Fluorescence Emitter Based on Ladder-Thiaborin Exhibiting Narrowband Sky-Blue Emission with Accelerated Reverse Intersystem Crossing. *Angew. Chem. Int. Ed.* **60**, 20280-20285 (2021).

80 Park, I. S., Yang, M., Shibata, H., Amanokura, N. & Yasuda, T. Achieving Ultimate Narrowband and Ultrapure Blue Organic Light-Emitting Diodes Based on Polycyclo-Heteraborin Multi-Resonance Delayed-Fluorescence Emitters. *Adv. Mater.* **34**, e2107951 (2022).

81 Park, I. S., Min, H. & Yasuda, T. Ultrafast Triplet-Singlet Exciton Interconversion in Narrowband Blue Organoboron Emitters Doped with Heavy Chalcogens. *Angew. Chem. Int. Ed.*

61, e202205684 (2022).

82 Li, Q. *et al.* Boron-, Sulfur- and Nitrogen-Doped Polycyclic Aromatic Hydrocarbon Multiple Resonance Emitters for Narrow-Band Blue Emission. *Chem. Euro. J.* **28**, e202104214 (2022).

83 Li, Q. *et al.* Selenium-Doped Polycyclic Aromatic Hydrocarbon Multiresonance Emitters with Fast Reverse Intersystem Crossing for Narrowband Blue Emission. *ACS Appl. Mater. Interfaces* **14**, 49995–50003 (2022).

84 Hua, T. *et al.* Heavy-atom effect promotes multi-resonance thermally activated delayed fluorescence. *Chem. Eng. J.* **426**, 131169 (2021).

85 Hu, Y. X. *et al.* Efficient selenium-integrated TADF OLEDs with reduced roll-off. *Nat. Photonics.* **16**, 803-810 (2022).

86 Wang, J. *et al.* Metal-Perturbed Multi-Resonance TADF Emitter Enables High-efficiency and Ultralow Efficiency Roll-off Non-Sensitized OLEDs with Pure Green Gamut. *Adv. Mater.* **35**, 2208378 (2023).

87 Yang, W. *et al.* Simple Double Hetero[5]helicenes Realize Highly Efficient and Narrowband Circularly Polarized Organic Light-Emitting Diodes. *CCS Chem.* **4**, 3463-3471 (2022).

88 Hu, Y. X. *et al.* Peripherally Heavy-Atom-Decorated Strategy Towards High-Performance Pure Green Electroluminescence with External Quantum Efficiency over 40% *Angew. Chem. Int. Ed.* **62**, e202302478 (2023).
