# Supplementary material for: Efficient, narrow-band, and stable electroluminescence from organoboron-nitrogen-carbonyl emitter
Source: Nat Commun. 2024 Jan 25;15:731. doi: 10.1038/s41467-024-44981-1 (PMC10810797; doi:10.1038/s41467-024-44981-1)
Supplement: Supplementary file 3 — Description of Additional Supplementary Files [file 41467_2024_44981_MOESM3_ESM.pdf]

Description of Additional Supplementary Files

File name: Supplementary Data 1

Description: Crystallographic Data of h-BNCO-1
